# EMTeC: A corpus of eye movements on machine-generated texts

**DOI:** 10.3758/s13428-025-02677-4

**Published:** 2025-06-03

**Authors:** Lena S. Bolliger, Patrick Haller, Isabelle C. R. Cretton, David R. Reich, Tannon Kew, Lena A. Jäger

**Affiliations:** 1 Department of Computational Linguistics, University of Zurich, Andreasstrasse 15, Zurich, 8050 Switzerland; 2 Department of Computer Science, University of Potsdam, An der Bahn 2, Potsdam, 14476 Germany

**Keywords:** Eye-tracking, Machine-generated, Reading, Decoding

## Abstract

The **E**ye movements on **M**achine-generated **Te**xts **C**orpus (EMTeC) is a naturalistic eye-movements-while-reading corpus of 107 native English speakers reading machine-generated texts. The texts are generated by three large language models using five different decoding strategies, and they fall into six different text-type categories. EMTeC entails the eye movement data at all stages of pre-processing, i.e., the raw coordinate data sampled at 2000 Hz, the fixation sequences, and the reading measures. It further provides both the original and a corrected version of the fixation sequences, accounting for vertical calibration drift. Moreover, the corpus includes the language models’ internals that underlie the generation of the stimulus texts: the transition scores, the attention scores, and the hidden states. The stimuli are annotated for a range of linguistic features both at text and at word level. We anticipate EMTeC to be utilized for a variety of use cases such as, but not restricted to, the investigation of reading behavior on machine-generated text and the impact of different decoding strategies; reading behavior on different text types; the development of new pre-processing, data filtering, and drift correction algorithms; the cognitive interpretability and enhancement of language models; and the assessment of the predictive power of surprisal and entropy for human reading times. The data at all stages of pre-processing, the model internals, and the code to reproduce the stimulus generation, data pre-processing, and analyses can be accessed via https://github.com/DiLi-Lab/EMTeC/.

## Introduction

Human eye movements in reading provide insight into the cognitive mechanisms involved in human language processing (Rayner, 1998) and reveal information about key properties and structures of the text being read (Rayner, 2009; Engbert et al., 2005; Reichle et al., 1998). As a consequence, the investigation and application of readers’ eye movements have experienced a great upswing within the past two decades across a variety of fields, including experimental and computational psycholinguistics, cognitive psychology, education science, natural language processing (NLP), and various areas of computer science, including human–computer interaction, robotics, and artificial intelligence.

### Psycholinguistic analysis of eye movements in reading

Within the field of psycholinguistics, most eye-tracking studies aim at investigating very clearly defined and usually theoretically motivated hypotheses about human reading and language comprehension processes. These hypotheses are frequently studied by using minimal pair stimuli that allow for a targeted manipulation of the linguistic construction under investigation, thereby enabling disentangling specific phenomena and isolate the ones that, based on clear theoretical assumptions, provide the answers to the hypotheses. This is an important and effective approach to studying linguistic constructions and their effect on reading behavior and the cognitive processes involved, especially as theoretically relevant constructions that allow for teasing apart competing theories are often complex and infrequently occurring in natural language.

However, research has also underlined the importance of studying language processing in a naturalistic setting (Demberg & Keller, 2008, 2019; Nastase et al., 2020): this entails moving away from minimal pair stimuli to ones that naturally occur in the language, such as newspaper articles or fictional texts. This is also accompanied by stimuli that extend beyond the sentence level and results in the possibility of examining reading behavior not only within a sentence but across sentence boundaries and entire paragraphs.

While minimal pair stimuli usually serve the investigation of lexical properties (e.g., word length, lexical frequency, or word predictability), or of syntactic and semantic processing at the sentence level (e.g., garden-path effects, similarity-based interference in syntactic dependency formation, grammatical illusions, or effects of local coherence), naturalistic stimuli at the paragraph level allow for studying different linguistic phenomena and discourse processing beyond sentence boundaries. These include research on co-reference resolution beyond the sentence boundary (Luo & Glass, 2018), the dependence of eye movement patterns on global text difficulty (Rayner et al., 2006), and the inference of passage-level text comprehension in adults (Reich et al., 2022; Prasse et al., 2024) and children (Joseph et al., n.d.). It is this expanding of controlled experiments with highly complex and constructed stimuli into the inclusion of naturalistic texts that has resulted in the usage of eye-tracking-while-reading data beyond the realm of psycholinguistics.

Another line of research that has been steadily growing involves surprisal theory (Hale, 2001; Levy, 2008). The notion of surprisal operationalizes the relationship between language and cognitive effort. More specifically, surprisal quantifies the predictability of a word given its context. Surprisal is defined as the negative log-probability of a word conditioned on its preceding linguistic and extra-linguistic context, and it has been argued to be proportional to the cognitive effort associated with the processing of this word, typically measured in terms of reading times. This correlation of surprisal with behavioral measurements in reading has been corroborated extensively (Demberg & Keller, 2008; Hoover et al., 2023; Pimentel et al., 2023; Shain, 2021, *interalia*).

### Leveraging eye movements in reading for technical purposes

In recent years, eye movements in reading have also been increasingly leveraged for more technical and application-oriented purposes and investigations. First, the accompaniment of the naturalistic stimuli by other (quasi-)experimental variables that are not tied to the stimuli themselves but to the readers enables both the statistical investigation as well as the inference of reader-specific properties from the eye movement data, such as the detection of dyslexia (Haller et al., 2022; Raatikainen et al., 2021) or the prediction of reading comprehension (Mézière et al., 2023; Prasse et al., 2024; Reich et al., 2022) and readers’ language proficiency (Berzak et al., 2018).

Second, eye-tracking-while-reading data has also been increasingly utilized within the field of natural language processing (NLP) for various use cases. On the one hand, it has been utilized to enhance the performance of language models (LMs) on a variety of downstream tasks (Deng et al., 2023, 2024; Hollenstein et al., 2020), such as sentiment analysis (Long et al., 2017; Mishra et al., 2017a; Tiwari et al., 2023; Yang & Hollenstein, 2023); named entity recognition (NER) (Hollenstein & Zhang, 2019); sentence compression and paraphrase generation (Sood et al., 2020); the generation of image captions (Takmaz et al., 2020); reading task classification (Hollenstein et al., 2021b); co-reference resolution (Cheri et al., 2016); natural language inference, word sense disambiguation and question answering (Wang et al., 2024); and improving machine comprehension (Malmaud et al., 2020).

On the other hand, eye movement data has also been employed to explore the cognitive plausibility of language models and the extent of their cognitive interpretability (Beinborn & Hollenstein, 2023; Keller, 2010). This line of research comprises the interpretation of neural attention and its alignment with human attention, quantified in terms of reading times (Bensemann et al., 2022; Eberle et al., 2022; Sood et al., 2020); the evaluation of language models’ abilities to predict human reading behavior (Hollenstein et al., 2021a, 2022b; Merkx & Frank, 2021); and the assessment of the alignment of importance attributed to words in a sentence between models and humans (Hollenstein & Beinborn, 2021).

Third, with the advent of neural language models, the investigation of surprisal theory has also gained new momentum. Eye-tracking-while-reading data is now not only used to investigate the predictability effects as postulated by surprisal theory, but they are also employed as indicators of how well surprisal can be estimated in the first place. Since surprisal presupposes access to the probabilities of words given their context and thus to the true probability distribution over the vocabulary, which is unknown in practice, it can only ever be approximated. Earlier approaches in this area aimed to measure surprisal using *n*-gram models (Fossum & Levy, 2012; Jurafsky & Martin, 2000; Mitchell et al., 2010) or Hierarchical Hidden Markov models (Wu et al., 2010) to approximate surprisal. Others have employed probabilistic context-free grammars (PCFGs), which are phrase structure grammars augmented with probabilities on the expansion rules (Hale, 2003, 2006; Roark et al., 2009). Surprisal can also be empirically measured by means of Cloze tasks (Wilson, 1953; Smith & Levy, 2013) or sentence completion tasks (Jäger et al., 2015). These methods are based on experiments with human participants during which they are given a context and have to guess the next or missing word. The probabilities are estimated as the proportion of subjects correctly guessing the word based on the context. More recent research has turned to the utilization of language models for the estimation of surprisal, such as long short-term memory neural networks (Goodkind & Bicknell, 2018).

Since the advent of the Transformer architecture (Vaswani et al., 2017), decoder-based autoregressive language models such as GPT-2 (Radford et al., 2019) have been a popular choice. This has initiated a parallel line of research into surprisal with a focus on which language models have the highest predictive power, i.e., which language model estimates surprisal in a way such that it has the best fit on human reading times. Studies have shown that there is a relationship between the predictive power of a language model and both its size in terms of parameters (Oh & Schuler, 2023b) and the size (and compositionality) of the data it has been trained on (Oh & Schuler, 2023a). Additionally, Haller et al. (2024) also show that the predictive power differs with respect to the cognitive and linguistic capacities of the readers for which the reading times are predicted, i.e., the predictive power hinges on whether readers have demonstrated high (or low, respectively) cognitive control, working memory, and reading fluency in psychometric assessment tests.

Finally, there has been an upsurge of machine learning-based generative models of human eye movements in recent years. While cognitive models of eye movements implement theories of reading, such as the E-Z Reader model (Reichle et al., 1998), the Über-Reader model (Veldre et al., 2020), or the SWIFT model (Engbert et al., 2005), these more recent models are purely data-driven. They simulate human eye movements on new texts that are similar to the ones they have been trained on, with some predicting fixation sequences (Bolliger et al., 2023; Deng et al., 2023; Hahn & Keller, 2016; Nilsson & Nivre, 2009, 2011; Wang et al., 2019) and others raw gaze coordinates (Prasse et al., 2023, 2024). For training such models, it is crucial to have a wide range of eye movement corpora available that span both different languages as well as different text types and linguistic characteristics.

### Reading as a cultural skill: The focus on what is being read

Eye movements in reading leveraged to investigate human language processing require – given the very nature of the research hypotheses – to place the focus on the experimental stimuli themselves, as many psycholinguistic premises can only be answered by manipulating said stimuli. However, the question of *what* is being read can also be approached in more general terms. Reading is both a cultural skill as well as a socio-cognitive process; it is not only an intellectual activity that contributes to problem-solving skills and the evolution of independent and critical thought processes (Sudha & Harinarayana, 2008), but it is also the means by which people learn culturally appropriate ways of engaging with the written texts and consume culturally relevant and appropriate information and values (Bloome, 1985). As such, what is being read – both the contents and their origins – is of importance on a level beyond the individual reader. The advent of large language models (LLMs) such as Gemini (Anil et al., 2024), Llama 2 (Touvron et al., 2023), and GPT-4 (OpenAI et al., 2024) has enabled the generation of texts that are not only grammatically correct but also highly persuasive and nearly indistinguishable from human-written material (Kumarage et al., 2024). Although the utility of these models cannot be denied, having been adopted in various domains such as journalism and academia, they do not come without ethical challenges. The combination of generating coherent text and the absence of consciousness and a real author persona can erode public trust and distort societal perceptions (Chakraborty et al., 2023); the true origin of such generated texts remains unknown to the readers. This is exacerbated by the intuition that humans spend their lives developing a bias towards assuming that well-written and eloquent texts are trustworthy. LLMs reduce this to absurdity, as they bring the form to perfection while the content is merely a by-product of their training. The European Union has recently adopted the Artificial Intelligence Act^1^, according to which the labeling of AI-generated content is mandatory, a law which is overall very difficult to enforce.

With reading being such a central component to both how values and information are distributed within a society as well as how said society is perceived by readers, it is imperative to investigate more closely how readers engage with texts generated by language models. As such, it might not only be relevant to inspect the output of these models, i.e., the texts they generate, and how people read those, but it might also be insightful to research *how* the models generate those texts and how different generation strategies affect reading behavior. This would entail looking into how different decoding strategies, i.e., different ways of modulating the probability distributions over the next words to be generated, affect the output texts and the way readers engage with them.

### Introducing EMTeC

In this paper, we present EMTeC, the **E**ye movements on **M**achine-generated **Te**xts **C**orpus, a naturalistic corpus of native English speakers’ eye movements in reading machine-generated texts of different genres. EMTeC is a clear departure from previous eye movements in reading corpora, as the experimental stimuli were generated with three different large language models (LLMs) of different size and from different model families, and that each LLM generated texts belonging to six different text types using five different neural decoding strategies.

Moreover, we not only release the eye movement data and the experimental stimuli, but we additionally provide the LLMs’ internals: the transition scores, attention scores, and hidden states for each generated text. We prompted the models to generate a series of texts of six different text types (e.g., fiction, poetry, summarization, ...). For the resulting stimulus corpus we created comprehensive text- and word-level annotations, such as surprisal estimates, text difficulty metrics, part-of-speech tags, dependency tags, and more. Moreover, each stimulus is accompanied by a comprehension question and two rating questions, one on subjective text difficulty and one on engagement with the text, which are answered by the participants.

In order to promote transparency, reproducibility, and reusability of EMTeC, we not only release the eye-movement data at its final processed stage but also all intermediate steps of pre-processing. More specifically, this comprises the raw data consisting of coordinate-timestamp samples, the fixation sequence data (scanpaths) extracted from the raw data by means of a gaze-event detection algorithm, and the word-level reading measures data computed from the fixation sequences. Moreover, we provide both an uncorrected and a corrected version of the data. In the latter, possible vertical drifts due to calibration degradation in the fixation data has been manually corrected by reassigning fixations that have been mapped to the wrong area of interest (the wrong word) to the correct one. We make all code available in a reproducible format, which includes the implementations of the stimulus generation, the text- and word-level annotation, the drift correction pipeline, and all stages of eye-tracking data pre-processing.

In summary, EMTeC offers these main features:

- The texts are machine-generated by LLMs of different sizes and different families using five of the most common decoding strategies;
- The texts belong to six different text types (non-fiction (argument and description), fiction (story and dialog), poetry, summarization, news article, keyword text)
- We release all LLM internals, i.e., the transition scores, attention scores, and hidden states;
- We release the eye movement data in all stages of pre-processing, i.e., raw coordinate data, fixation sequences, and reading measures;
- We release both the corrected and uncorrected eye movement data;
- We release text- and word-level annotations (surprisal, PoS tags, dependency tags, readability scores, ...)
- All code is made publicly available, allowing for transparency over the entire pipeline from stimulus generation to pre- and post-processing of the eye movement data.

## Background

This section aims to provide background information on topics or concepts relevant to understanding why and how EMTeC was created and curated. First, we discuss what eye movements in reading are and what kind of information about humans, texts, and the cognitive mechanisms involved in language processing can be gleaned from them. Second, we outline what autoregressive language models are. This is not only relevant for understanding how the stimuli in EMTeC are generated but is also relevant in a more general way, as autoregressive language models are increasingly employed in investigating human reading behavior, for instance to estimate surprisal. Moreover, this section describes how the decoding strategies used for text generation are defined and outlines what instruction-tuned language models are, thereby motivating the choice of LLMs used for creating this corpus’s stimuli.

### Eye movements in reading

When humans read text, their eyes perform two main actions: during *fixations*, the eye remains relatively still on one point of focus and the brain obtains visual input; and during *saccades*, which are ballistic relocation movements, the eye jumps from one fixation to another and is “blind”. While average fixations during reading last about 200–250 ms, saccades are very rapid movements of 20–80 ms that drive the eye about 7–9 letter spaces further in the text (Rayner, 1998, 2009).

Reading is a complex task in which a variety of cognitive processes are involved. Different words are fixated for longer or shorter times, or they are skipped entirely (Liversedge & Findlay, 2000). Many models have been trying either to explain specific aspects of the reading process, such as word identification or syntactic parsing, or to investigate how these subcomponents of language processing in reading (like word identification) in conjunction with other constraints (memory, visual acuity) guide readers’ eyes (Rayner & Reichle, 2010). One of the most important models is the E-Z Reader model (Reichle et al., 1998), which outlines how eye movements in reading are dependent on lexical factors: features such as word frequency or predictability affect the duration of a fixation and whether or not a word is skipped. The other important model is the SWIFT model (Engbert et al., 2005), which posits that variables like word frequency influence fixation durations indirectly by inhibiting a random timer that determines the moment-to-moment decisions about when to initiate a saccade.

Eye movements in reading thus reveal insight into both the cognitive processes underlying human language comprehension as well as linguistic properties of the text (Radach & Kennedy, 2004). On the one hand, they exhibit between-subject idiosyncrasies which remain consistent within-subject across different tasks (Bargary et al., 2017) and can even be used to infer subject identity (Jäger et al., 2020). On the other hand, being driven by an intricate interplay – both voluntary and involuntary – between cognition, attention, and oculomotor control, they can be indicative of a wide range of cognitive abilities and processes, such as cognitive load (Delgado & Salmerón, 2022), working memory (Huang et al., 2022; Indrarathne & Kormos, 2018), attention (Rodrigue et al., 2015), and dyslexia (Raatikainen et al., 2021; Haller et al., 2022).

### Autoregressive language models

Autoregressive language models (LMs), also denoted $P_\theta$, where $\theta$ are the model parameters, are a class of probabilistic models that assign a probability to each word in a vocabulary in order to predict the next word in a sequence conditioned on previous context.^2^

Formally, given a sequence of words as context, ${\textbf {w}}_{<t} := (w_1, \ldots , w_{t-1})$, the model computes the conditional probability of all possible words $w_t$ in a predefined vocabulary *V* at each generation step *t*:

1 $$\begin{aligned} P_\theta (w_t | {\textbf {w}}_{<t}). \end{aligned}$$

These probabilities denote the likelihood of moving from one word to the next and are thus often referred to as transition scores.

Current state-of-the-art LMs (e.g. Brown et al., 2020; Touvron et al., 2023) are based on decoder-only transformers (Liu et al., 2018). These comprise a stack of transformer blocks (Vaswani et al., 2017) with a language modeling head (i.e., a linear layer with an output dimension equal to the size of the vocabulary). As input, the model takes a sequence of word embeddings augmented with positional information representing the current context. This representation is passed through multiple layers of transformer blocks, comprising multi-head self-attention, linear projection, and normalization operations together with the addition of a residual stream to produce a sequence of informative and contextualized hidden states. The final hidden state of the last context word is then passed through a linear layer to produce a score vector over the vocabulary. These scores are unnormalized and commonly referred to as logits. Applying the softmax function to this set of scores converts it into a valid probability distribution over the vocabulary, where all values are positive and sum to 1.

Transformer-based autoregressive LMs can be pre-trained effectively at scale and on very large corpora using the simple self-supervised learning objective of next-word prediction. Given a context sequence from the training corpus, the model is tasked with predicting the most likely continuation by computing a probability distribution over all possible vocabulary items. Based on this prediction, the loss is computed as cross-entropy between the predicted distribution and the true distribution of the next word, which is backpropagated through the model to update the parameters such that the overall loss is minimized. Doing this repeatedly and for all possible context sequences in a large and diverse training corpus allows neural models to learn the distribution of words as well as nuanced syntactic (Blevins et al., 2018; Hewitt & Manning, 2019; Manning et al., 2020; Wei et al., 2021) and semantic patterns (Merrill et al., 2024; Rogers et al., 2020; Vulić et al., 2020; Zhang et al., 2021) in the language.

Once trained, a decoder-based language model can be used to generate text autoregressively, meaning that the input context is continuously updated with each new output prediction. This iterative process of predicting the next word in a sequence continues until a special end-of-sequence ([EOS]) token is generated or until a maximum length is reached.

#### Instruction-tuned language models

Following semi-supervised pre-training, autoregressive language models are typically fine-tuned using supervised learning techniques in order to adapt them for a particular task. Recently, general-purpose instruction tuning has emerged as a promising approach for deriving highly capable instruction-following models with impressive generalization abilities (Chung et al., 2024; Iyer et al., 2023; Ouyang et al., 2022; Sanh et al., 2022).^3^ This step employs the same learning strategy as pre-training (i.e., minimizing the cross-entropy loss on the next-token prediction task) but applies it to supervised training data in the form of templated instruction–response pairs. Here, the instruction is considered as the input context and an appropriate response is treated as the target sequence that the model should generate. Given adequate supervised instruction tuning, a language model can be prompted using natural language instructions to perform a diverse array of text-based tasks, such as open-ended story generation, question answering, and summarization, among others.

#### Decoding strategies

To generate text from an autoregressive LM, a decoding strategy is used to select which word is generated next, given the probability distribution over all words in the vocabulary computed by the LM at each timestep. Numerous decoding strategies have been proposed, each with their own trade-offs in terms of speed and quality, as well as inherent biases that they impose on the generation process (Eikema & Aziz, 2020; Holtzman et al., 2020; Meister et al., 2020). The most popular strategies can be placed into two categories: likelihood-maximization methods, which encompass greedy search and beam search; and stochastic methods, which involve ancestral sampling, top-*k* sampling, and top-*p* sampling.

##### Likelihood-maximization strategies

Likelihood-maximization strategies aim to approximate maximum *a posteriori* (MAP) search over all possible output sequences conditioned on the context under the model. This approximation is necessary since exact MAP decoding is intractable given the exponentially large search space for any given text sequence.

*Greedy decoding* is a relatively simple method that selects the word $w_{t}$ out of the vocabulary $V$ with the highest probability at each generation step $t$:

2 $$\begin{aligned} w_{t} = \underset{w \in V}{arg\,max}\ P_{\theta } (w|{\textbf {w}}_{< t}). \end{aligned}$$

While fast, greedy decoding has been found to result in dull and repetitive texts, often exhibiting degenerate word repetitions and other disfluencies (Holtzman et al., 2020).

*Beam search* is a more sophisticated strategy that aims at alleviating the issue of making locally optimal decisions in constructing a sequence by maintaining multiple possible hypotheses. At each generation step, each hypothesis is expanded by considering the *l* most likely words, resulting in $l^2$ possible continuations (i.e., *beams*). Hypotheses are then pruned to keep only the *l* most likely sequences and the process is repeated until all hypotheses have reached the end-of-sequence token or a maximum length, at which point the sequence with the highest probability is returned as the final output.^4^ More formally, consider the *l* solutions $S_{t-1}$ for beam search at time step $t-1$: $S_{[t-1]}=\left\{ \textbf{w}_{<t,1}, \ldots , \textbf{w}_{<t, l}\right\}$, where $\textbf{w}_{<t,k}$ are the top *l* highest scoring continuations. At time step *t*, beam search considers all possible continuations $w\in V$ and retains the *l* highest, i.e.,

3 $$\begin{aligned} S_t&= arg\,max_{w_1, \ldots , w_l\in V}\sum _{i\in [l]}P_\theta (\textbf{w}_{< [t+1],i})\end{aligned}$$

4 $$\begin{aligned}&= arg\,max_{w_1, \ldots , w_l\in V}\sum _{i\in [l]}\sum _{w_i\in V}P_\theta (w_i\mid \textbf{w}_{< t,i}). \end{aligned}$$

While beam search has long been the de facto choice of decoding strategies in tasks such as machine translation and speech recognition (Koehn et al., 2003; Och et al., 1999), it often tends to produce less diverse outputs (Vijayakumar et al., 2018), making it less suitable for more creative, open-ended style generation tasks. Additionally, it is slower and more memory-intensive than other decoding strategies due to having to keep track of multiple hypotheses.

##### Stochastic strategies

In order to improve the diversity of generated text, a number of strategies rely on stochastic sampling from the model’s output distribution.

*Ancestral sampling* randomly draws $w_t$ from the model’s full probability distribution over the vocabulary $V$ at each generation step:

5 $$\begin{aligned} {w}_{t} \sim P_{\theta }(w\mid {\textbf {w}}_{< t}). \end{aligned}$$

While this sampling strategy allows for constructing a text sequence that is faithful to the model, it is susceptible to errors and biases present in the estimated distribution, particularly in the long tail of low-probability words (Freitag et al., 2023). To mitigate this, other sampling strategies truncate the output distribution to restrict the selection to high-probability words.

*Top-k sampling* (Fan et al., 2018) considers only the *k* most likely candidates at each time step: the vocabulary $V$ is subset to $V_{k,t}$ such that $V_{k,t}$ consists of the $k$ most likely words at the current generation step $t$:

6 $$\begin{aligned} V_{k,t} = \underset{V' \subset V: |V'| = k }{arg\,max}\ \hspace{.5em}\underset{w \in V'}{\sum }\ P_\theta \left( w\mid {\textbf {w}}_{<t}\right) . \end{aligned}$$

Here, $V' \subset V: |V'| = k$ denotes all possible subsets of the vocabulary *V* with cardinality *k*. $w_{t}$ is then drawn from the renormalized top-*k* probability distribution for tokens in $V_{k,t}$ and 0 otherwise:

7 $$\begin{aligned} P(w_t\mid {\textbf {w}}_{<t}) = {\left\{ \begin{array}{ll} \frac{P_{\theta }(w\mid {\textbf {w}}_{<t})}{\underset{w \in V_{k,t}}{\sum } P_\theta (w\mid {\textbf {w}}_{<t})} & \text {if } w \in V_{k,t}, \\ 0 & \text {otherwise}. \end{array}\right. } \end{aligned}$$

In this strategy, *k* is set to a fixed value between $\left[ 1, \left| V\right| \right]$, with popular values typically ranging from 10 to 50, depending on the task.^5^

*Top-p (or nucleus) sampling* (Holtzman et al., 2020) constructs the minimal subset of possible candidates by keeping only those words whose cumulative probability mass is above a certain threshold *p*. This subset denotes the *nucleus* and often contains anywhere between 1 and 1000 words (Holtzman et al., 2020). Thus, in contrast to top-*k*, the size of the considered subset is dynamic given the model’s output distribution at each timestep. The vocabulary $V$ is subset to the nucleus $V_{p,t}$, formalized as

8 $$\begin{aligned} V_{p,t}=min\left| \left\{ w\in V: \sum _{w\in V}P_\theta (w\mid \textbf{w}_{<t})>p\right\} \right| . \end{aligned}$$

Again, $w_{t}$ is then drawn with probability proportional to the original probability distribution for tokens in the nucleus $V_{p,t}$ and 0 otherwise:

9 $$\begin{aligned} P(w_t\mid {\textbf {w}}_{<t}) = {\left\{ \begin{array}{ll} \frac{P_{\theta }(w\mid {\textbf {w}}_{<t})}{\underset{w \in V_{p,t}}{\sum } P_\theta (w\mid {\textbf {w}}_{<t})} & \text {if } w \in V_{p,t}, \\ 0 & \text {otherwise}. \end{array}\right. } \end{aligned}$$

Temperature scaling can also be used to modify the sharpness of the model’s next-token probability distribution when computing it over the set of output logits $z_i$

10 $$\begin{aligned} q_i = \frac{\text {exp}(z_i / \tau )}{\sum _{j=1} \text {exp}(z_j / \tau )}. \end{aligned}$$

Higher temperatures ($\tau > 1$) flatten the distribution and increase stochasticity for all sampling strategies, while lower temperatures ($\tau < 1$) sharpen the distribution, effectively decreasing stochasticity.

## Related work

The following section outlines an overview of existing corpora of eye movements in reading. Most of the existing eye movements in reading datasets consist of minimal pair stimuli; we will only review corpora comprising naturalistic or at least semi-naturalistic (semi-constructed) stimuli that are multi-purpose and do not follow a minimal pair design.

Naturalistic reading entails the reading of texts that occur or could occur naturally in the language. Naturalistic stimuli do not contain experimentally manipulated syntactic structures or lexical items that target a specific research hypothesis, as opposed to constructed or partially constructed stimuli. Moreover, naturalistic reading does not include task solving, time constraints, or other constraints. such as restricting the preview of the subsequent word or phrase.

These naturalistic eye movement corpora in reading can be further divided according to the text presented, i.e., whether they are single-sentence or entire paragraphs, and according to whether they are mono- or multilingual. In this section, we will only focus on passage-level reading; for an overview of single-sentence corpora, please refer to Appendix A.

Monolingual eye movement corpora on the passage level consist predominantly for English. For instance, the Provo corpus (Luke & Christianson, 2018) contains the data of 84 participants reading 55 English texts from a variety of sources, and GazeBase (Griffith et al., 2021) provides the data of 322 subjects reading up to 18 passages of a poem over the course of three years. Examples of eye movement corpora in other languages include InDiCo (Haller et al., 2023), which comprises German naturalistic reading data from four experimental sessions from each participant; PoTeC (Jakobi et al., 2024), comprising data from German native speakers reading textbook passages from two different domains (biology and physics); and RastrOS (Leal et al., 2022), comprising 37 subjects reading Portuguese texts. For the entire list of monolingual corpora, please refer to Table 1.

There also exists a range of multilingual eye-tracking-while-reading corpora on the passage level. For instance, the Multilingual Eye-Movements Corpus (MECO-L1) (Siegelman et al., 2022) contains 13 languages with around 50 subjects per language who read 12 encyclopedic texts; and the Ghent Eye-Tracking Corpus (GECO) (Cop et al., 2017) had 14 English monolinguals read a novel in English and 19 bilinguals read half of the novel in English and the other half in Dutch. For the full list, please refer to Table 1.

**Table 1:** Overview of naturalistic eye-tracking-while-reading corpora

Corpus Name	Language(s)	Details
*Monolingual Corpora*		
Provo Corpus (Luke & Christianson, 2018)	English	84 participants reading 55 texts
GazeBase (Griffith et al., 2021)	English	322 participants, 18 texts
InDiCo (Haller et al., 2023)	German	Data from four sessions per participant with psychometric test scores.
PoTeC (Jakobi et al., 2024)	German	German speakers reading physics and biology texts, categorized by expertise.
MECO-L2 (Kuperman et al., 2023)	English	543 non-native speakers from 12 L1 backgrounds reading 12 texts.
PopSci Corpus (Wolfer et al., 2013)	German	17 participants reading 16 popular science texts.
OneStopQA (Malmaud et al., 2020)	English	296 participants reading ten articles with pre/post comprehension questions.
RastrOS (Leal et al., 2022)	Portuguese	37 participants reading 50 paragraphs.
Mental Simulation Corpus (Mak & Willems, 2019)	Dutch	102 participants reading 3 short stories and answering questions.
SB-SAT (Ahn, Kelton, Balasubramanian, and Zelinsky, 2020)	English	95 participants reading 4 SAT passages, rating subjective difficulty.
CopCo (Hollenstein et al., 2022a)	Danish	22 native, 19 dyslexic, and 10 L2 speakers reading 20 speech manuscripts.
CopCo L2 (Reich et al., 2024)	Danish	Extension of CopCo with L2 recordings.
Parafoveal Processing Corpus (Parker et al., 2017)	English	Corpus focusing on parafoveal processing, manipulating word placement inside/outside parafoveal view.
MAQ-RC (Sood et al., 2020)	English	23 participants reading 32 movie plots and answering comprehension questions.
CFILT Datasets (Cheri et al., 2016; Mishra et al., 2017b; Mathias et al., 2018, 2020)	English	Includes co-reference resolution, scanpaths, text quality, and essay grading datasets using eye-tracking data.
Chinese article summarization (Yi et al., 2020)	Chinese	50 participants, 100 Chinese articles
SummarEyes (Taieb-Maimon et al., 2023)	English	80 participants reading and summarizing 4 texts.
*Multilingual Corpora*		
MECO-L1 (Siegelman et al., 2022)	13 languages	45–55 participants per language reading 12 encyclopedic texts.
GECO (Cop et al., 2017)	English, Dutch	14 monolinguals and 19 bilinguals reading a novel in English and Dutch.
GECO-CN (Sui et al., 2023)	Chinese, English	30 participants reading a novel in Chinese and English.
WebQAmGaze (Ribeiro et al., 2024)	4 languages	Recordings captured using a webcam.
Dundee (Kennedy et al., 2003)	English, French	10 participants, newspaper extracts
*Corpora on Machine-Generated Texts*		
GECO-MT (Colman et al., 2022)	Dutch	20 participants, 672 human-translated and 672 machine-translated texts

To the best of our knowledge, there exists only a single corpus that comprises eye-tracking-while-reading data on machine-generated texts: the Ghent Eye-Tracking Corpus of Machine Translation (GECO-MT) (Colman et al., 2022). The purpose of this corpus is to compare human and machine translations, and it contains eye movement data on both a human and a machine translation of Agatha Christie’s novel *The Mysterious Affair at Styles*. Twenty Dutch-speaking university students participated in the experiment. The machine translations were generated with Google Translate, deployed in May 2019. They do not provide any model-related information or model internals, which is an inherent limitation of using closed-source services.

## Stimuli

This section outlines the generation of the stimuli, including the steps taken to post-process and annotate them with linguistic annotation layers. Additionally, we also describe the generation of the comprehension questions related to the stimuli and provide descriptive statistics of the experimental items. All stimuli can be found in the file data/stimuli.csv, which not only contains the stimuli, the prompts, the comprehension questions, and possible answers as presented in the eye-tracking experiment but also all stages of post-processing, including unique word and token identifiers that allow mapping between sub-word tokens and word tokens and between sub-word tokens and the sub-word token-level transition scores, attention scores, and hidden states. A detailed description of all columns in the stimuli file is provided in file data/stimuli_columns_descriptions.csv.

### LLMs for stimuli generation

To generate the reading stimuli, we use three open-weight large language models (LLMs) of different size and from different language model families: Phi-2 (Javaheripi et al., 2023); Mistral 7B Instruct (Jiang et al., 2023); and WizardLM 13B (Xu et al., 2023).

Phi-2 (Javaheripi et al., 2023) is a 2.7-billion-parameter model pre-trained on 1.4T tokens sourced from NLP and coding datasets available on the web as well as high-quality synthetic data.^6^ Despite not having undergone explicit instruction tuning, this model is still adept at following instructions and responding to prompts, presumably due to the nature of its pre-training data.

Mistral (Jiang et al., 2023) is a 7-billion-parameter model that uses a modified attention mechanism to facilitate improved efficiency and better handling of longer sequences.^7^ While there is little information available on the pre-training data used for Mistral, the model was instruction-tuned using publicly available instruction tuning datasets.

WizardLM (Xu et al., 2023) is an instruction-tuned variant of the 13-billion-parameter Llama 2 model (Touvron et al., 2023).^8^ This model was tuned using a diverse set of synthetic and real-world instruction-following examples.

Notably, in selecting LLMs for our task, we refrain from using models that have undergone additional post-training stages such as reinforcement learning from human feedback (RLHF) (Ouyang et al., 2022). RLHF is a policy-based approach, which involves generating candidate responses to an input prompt (i.e., referred to as the rollout), scoring the generated responses with a reward model, and then updating the policy (in this case, the model) accordingly. Related work does not explicitly state which decoding strategies are used during the rollout phase (Touvron et al., 2023; Ouyang et al., 2022; Stiennon et al., 2020). However, we hypothesize that the selection of a certain decoding strategy used during policy-based training may introduce potential biases towards certain types of decoding strategies. To mitigate this, we avoid using models trained with RLHF. Instead, we restrict our model selection to pre-trained and instruction-tuned models, which are trained with a standard maximum likelihood estimation (MLE) objective. In Appendix B.1, we provide a detailed justification for why we selected these particular instruction-tuned models.

For each model, we generate five output texts for a given prompt by employing the five different decoding strategies described in §2.2.2: greedy search, beam search^9^, ancestral sampling, top-*k* sampling (Fan et al., 2018), and top-*p* sampling (Holtzman et al., 2020).

#### Prompts

Our prompts are designed to cover different tasks and distinct text types. This ensures that the output is as varied as possible to allow for a high generalizability of the analyses that can be done with the corpus. The two main generation tasks are *unconstrained* and *constrained* text generation: during the *unconstrained* generation, the model is not restricted with respect to its output, meaning it can be neither “right” nor “wrong”.

The *unconstrained* prompts are further divided into *non-fiction*, prompting the model to either write an argument on a certain topic or provide a description of a topic or scene; *fiction*, prompting the model to either write a short story or a dialogue between two characters; and *poetry*, prompting the model to compose a poem.

In the *constrained* setting, the model was restricted pertaining to its output, i.e., it was clear to some extent what the model was expected to produce and there is a way for the model to hallucinate (when the model produces output that sounds plausible but is either fabricated or inaccurate). *Constrained* prompts can be further divided into *summarization*, prompting the model to provide a summarization of an input text; *synopsis*, where the model is asked to write a news article based on a synopsis it is provided with; and *keyword text generation*, comprising the model being given five keywords and being prompted to compose a text based on them. An overview of the different prompt types can be seen in Table 2, and the full list of prompts can be found in the file data/stimuli.csv.

**Table 2:** The prompt types used and the number of prompts

Type	Task	Sub-type	Number	Description
Unconstrained	Non-fiction	Argument	6	The generated text should present an argument on a certain topic.
		Description	5	The generated text describes a topic or scene.
	Fiction	Story	3	The model is prompted to write a story.
		Dialogue	3	The model is prompted to write a dialogue between two characters.
	Poetry		4	The model is prompted to write a poem.
Constrained	Summarization		8	The model is asked to generate a summary of a provided text.
	Synopsis		7	The model is asked to write a news article based on a synopsis.
	Keyword text		6	The model is given keywords and has to compose a text based on them.

Instruction-tuning typically involves formatting training examples with a pre-defined prompt template. These prompt templates often differ across models, as shown in Fig. 7. In order to construct the full prompts used to generate the stimuli from each model, we fill the model-specific prompt templates accordingly.

##### Item selection

Our corpus consists of texts generated from 42 different prompts, which were selected based on criteria outlined further below. To conduct the selection, we crafted a total of 106 prompts for which the models generated outputs with all investigated decoding strategies. From the model outputs, we then selected 42 items that resulted from 42 prompts, each item consisting of 14 conditions, one for each combination of model and decoding strategy. The selection criteria ensured that all the 14 conditions corresponding to a single item were suitable to be presented to the participants in an eye-tracking experiment who were unaware of the fact that the stimuli were machine-generated. Items were excluded if, for at least one condition, the model (i) responded only with one word or one short sentence (ii) the model repeated the question or the prompt (iii) the model was self-referential (e.g., *As an AI model, ...*), or (iv) there was an *I*-narrator. We further selected the items in a way that the different text types are balanced.

#### Generation parameters and experiment

When generating texts with language models, there is a range of hyper-parameters that have to be determined a priori which control the generation process and shape the model output. In order to be able to present one text on a single screen during the eye-tracking experiment (as opposed to splitting it across several screens) while maintaining a font size that is not only legible but also big enough to account for a facilitated attribution of fixations to areas of interest (Holmqvist et al., 2011), we set the maximal number of generated tokens to 150^10^. For sampling-based decoding strategies, we used a temperature of 0.8. We set $k=50$ for top-*k* sampling, and $p=0.9$ for top-*p* sampling.

These settings aim to allow for a sufficient degree of creativity while maintaining coherency and fluency. For beam search, we set the number of beams (*l*) to 4 and did not apply early stopping. We refrained from using larger beam sizes due to the known deficiencies of beam search (Koehn & Knowles, 2017), which relate an increase in beam size to a decrease in output quality, as well as the additional computational overhead involved with keeping track of a larger number of hypotheses. For a detailed account on the decoding parameters, please refer back to Section “Decoding strategies”.

We loaded the models across five NVIDIA GeForce RTX 3090 GPUs for generation. All models are implemented in PyTorch (Paszke et al., 2019) and loaded from Huggingface (Wolf et al., 2020).

#### Post-processing of generated stimuli texts

##### Truncation

In order to present participants only with complete texts so as not to bias their eye movements, we removed incomplete sentences from the generated model outputs, resulting from the models attempting to exhaust the full 150 tokens set as a maximum number of tokens to generate. We further removed EOS tokens and trailing white-space and newline characters. Regarding poems, we additionally restricted the number of newline characters to nine to ensure that the poems fit on the presentation screen, with each poem typically consisting of two stanzas of four verses each, separated by a newline. The final texts as they were presented in the eye-tracking experiments can be found in the column gen_seq_trunc in the stimuli.csv file.

##### Cleaning the data

With prospective analyses using eye movement data in mind, we post-processed the generated sequences and are releasing the different stages of this post-processing. Since the fixation data and reading measures are at the word level – i.e., areas of interest are determined by splitting a text at whitespace characters – data cleaning was performed to ensure that this mapping to word level can be executed correctly.

Data cleaning to ensure suitability for eye movement data analyses involves removing trailing and superfluous white-space characters, punctuation marks, accounting for otherwise problematic output and ultimately mapping the sub-word level model output to word-level. The data cleaning process consisted of the following pipeline:

1. Map the generated sub-word tokens to word IDs. This ensures that sub-word tokens belonging to the same word are grouped together and that transition and attention scores and hidden states can be mapped to the correct word.^11^ This allows for a proper one-to-one mapping between the words and the areas of interest as defined in the eye-tracking experiment.
2. Remove newlines and white-space characters, as they do not obtain fixations. This is done by attributing a unique token ID to every generated token, which also includes white-space characters, and then subsetting these token identifiers to those that will potentially have fixations on them. These token identifiers can then in turn be used to subset the transition scores, attention scores, and hidden states and discard those that do not refer to an area of interest.
3. We optionally included a further stage of post-processing that allows for removing sub-word tokens and scores referring to punctuation marks to allow for the possibility of not including the scores the language models produced for punctuation when computing word-level metrics such as surprisal or entropy.

The post-processing steps merely result in the correct subset of unique token identifiers that enable a correct mapping between areas of interest that contain fixations and reading measures and the generated tokens in case of analyses with the scores; they allow for accessing not only the original generated sub-word tokens and scores before post-processing, but also the truncated versions, the versions without white-space characters and newlines, and the versions without punctuation marks. The experimental stimuli presented in the experiment are acquired from the first step of post-processing, which is truncation; all white-space characters, newlines and punctuation marks are thus presented to the participants as the language models generated them.

### Lexical annotation

#### Annotation on Text Level

On the text level, we report the length of a text in number of words, the average word length in number of characters, the average Zipf frequency, and the average word frequency obtained from the wordfreq library.^12^ The frequency of a word is reported for words that appear at least once in 100 million words as a min-max normalized decimal between 0 and 1. The Zipf frequency of a word presents the lexical word frequency on a more human-friendly logarithmic scale and is the word’s base-10 logarithm of the number of times it appears in a billion words. The data for the wordfreq library stems from the Exquisite Corpus,^13^ which compiles eight different text domains (some of which come from different sources themselves): Wikipedia, Subtitles, News, Books, Web Text, Twitter, Reddit, and Miscellaneous. On the text level, the word frequency and the Zipf frequency values are averaged across all words in a text.

We further report eight different text readability metrics computed with the readability package^14^. They include the Flesch Reading Ease and the Flesch Kincaid Grade Level (Kincaid et al., 1975); the Gunning Fog Index (Gunning, 2004); the Coleman-Liau Index (Coleman & Liau, 1975); the Dale-Chall Readability Formula (Dale & Chall, 1948); the Automated Readability Index (Senter & Smith, 1967); the Linsear-Write Readability Metric (O’Hayre, 1966); and the Spache readability formula (Spache, 1953). Note that these metrics can only be computed above a certain text length threshold.

#### Annotation on Word Level

On word level, we report the word length in characters with and without punctuation; the part-of-speech, the dependency tag, the number of left and right dependents, and the distance to the head, all extracted with spaCy 15; whether or not a word is last in line as presented in the eye-tracking experiment; the word frequency and the Zipf frequency, again computed with the wordfreq library; and the surprisal (Hale, 2001; Levy, 2008), which is the negative log-probability of a word given its preceding context. Since the predictive power of surprisal on human reading times has been shown to differ depending on the language model from which it has been extracted  (Goodkind & Bicknell, 2018; Oh & Schuler, 2023a, b; Wilcox et al., 2023, 2020), we estimate surprisal from a range of different language models: GPT-2 *base* and *large* (Radford et al., 2019); OPT-350m and OPT-1.3b (Zhang et al., 2022); Llama-2-7b and Llama-2-13b (Touvron et al., 2023); Phi-2 (Javaheripi et al., 2023); Mistral 7b (Jiang et al., 2023); and Pythia-6.9b and Pythia-12b (Biderman et al., 2023). Moreover, we estimate surprisal and entropy for the stimulus text in two different ways: once the input to the above-mentioned language models is the generated stimulus text alone, and once the input is both the combination of prompt and generated stimulus text. The latter is provided for the option of having a consistent comparison with the transition scores, which are conditioned on the prompt as well.

### Comprehension question generation

For each of the 42 items in each of the 14 experimental conditions, we created a multiple-choice comprehension question with four possible answers. The purpose of these questions is to encourage participants to maintain their attention level and properly read the text, as opposed to merely skimming them and mindlessly clicking through the experiment. The comprehension questions were generated by prompting ChatGPT (Brown et al., 2020; OpenAI et al., 2024). The prompt template used is provided in Appendix B.3.

**Table 3:** Descriptive statistics across all experimental stimuli in *all conditions*

	Text length	Word length	Zipf freq.	Flesch	Surp. GPT-2
Mean	86.515	5.16	5.644	43.035	4.018
Std	20.675	0.519	0.229	22.631	3.77
Min	25.0	3.621	4.827	4.783	0.0
Max	130.0	6.66	6.406	108.073	30.897

To ensure the quality and correctness of the questions’ content, we manually validated all generated questions and answers and made minor edits to instances where any of the following issues were observed:

i) more than one possible answer was true;
ii) the length of the true answer differed from the length of the three false answers;
iii) the question included an entire phrase of the text verbatim; or
iv) the question did not refer to the text directly but to world knowledge about the topic of the text.

We further re-attributed the positions of correct answers such that their ultimate distribution (i.e., whether the correct answer was answer a, b, c, or d) is uniform.

### Descriptive statistics of the generated stimuli

We provide the descriptive statistics of a selection of the lexical characteristics of the stimuli. Table 3 depicts the overall average text length, the average word length, the average Zipf frequency, the average Flesch reading ease score, and the average surprisal value extracted from GPT-2 *base*.

**Table 4:** Descriptive statistics for *each model*

		Text length	Word length	Zipf freq.	Flesch	Surp. GPT-2
Phi-2	Mean	84.327	5.131	5.686	40.228	4.0
	Std	26.246	0.571	0.217	25.755	3.728
	Min	25.0	3.795	4.889	11.623	0.0
	Max	130.0	6.411	6.189	105.304	30.897
Mistral	Mean	89.995	5.155	5.643	44.775	3.935
	Std	19.287	0.462	0.234	20.18	3.714
	Min	37.0	3.621	4.943	5.949	0.0
	Max	124.0	6.66	6.406	100.731	30.897
Wizardlm	Mean	84.786	5.189	5.611	43.54	4.121
	Std	16.097	0.53	0.23	22.172	3.859
	Min	28.0	3.903	4.827	4.783	0.0
	Max	118.0	6.405	6.117	108.073	30.897

The same statistics can be found in Table 4, where they are averaged not across all stimuli but across those stimuli generated by one LLM. Mistral produces, on average, the longest texts, and while WizardLM and Phi-2 are equal with respect to text length, Phi-2 exhibits the greatest variability of all three models. According to the Flesch score, Phi-2 also appears to generate, on average, slightly easier texts than the other two models, although its standard deviation is again highest.

**Table 5:** Descriptive statistics for each *generation task*

		Text length	Word length	Zipf freq.	Flesch	Surp. GPT-2
*Unconstrained*						
Non-Fiction	Mean	96.247	5.276	5.706	41.635	3.703
	Std	16.071	0.412	0.182	15.482	3.378
	Min	44.0	3.901	5.139	12.497	0.0
	Max	127.0	6.217	6.189	91.995	27.205
Fiction	Mean	93.536	4.608	5.806	72.119	3.598
	Std	16.315	0.398	0.19	17.238	3.473
	Min	42.0	3.853	5.178	46.583	0.0
	Max	130.0	5.59	6.086	108.073	30.897
Poetry	Mean	57.518	4.493	5.628	50.667	5.743
	Std	11.623	0.365	0.311	33.947	4.412
	Min	37.0	3.621	4.943	51.959	0.0
	Max	84.0	5.25	6.406	91.445	30.897
*Constrained*						
Summarization	Mean	78.071	5.28	5.538	40.199	4.549
	Std	19.573	0.36	0.22	18.882	4.103
	Min	28.0	4.514	4.827	18.176	0.0
	Max	113.0	6.303	5.976	77.069	24.651
Synopsis	Mean	88.878	5.48	5.51	27.874	4.119
	Std	17.407	0.376	0.184	11.435	3.99
	Min	40.0	4.5	5.016	5.949	0.0
	Max	128.0	6.66	5.876	59.635	29.789
Keywords	Mean	89.488	5.413	5.677	32.898	3.604
	Std	20.497	0.409	0.188	14.306	3.541
	Min	25.0	4.649	5.259	4.783	0.0
	Max	124.0	6.411	6.048	62.19	29.315

In Table 5, the same lexical characteristics are displayed across text types. As to be expected, we observe a high degree of variability within the *unconstrained* text types, with *non-fiction* and *fiction* producing the longest texts and *poetry* the shortest. The *constrained* text types’ average text lengths are closer together in magnitude, with *summarization* producing, on average, the shortest texts, probably owing to the nature of the task. There are also discernible differences pertaining to Flesch reading ease: the *fiction* texts are, on average, the most difficult ones, followed by *poetry*, which exhibits the highest variability in difficulty; the articles generated based on an article *synopsis* are on average the easiest, according to the Flesch score. Regarding surprisal, the *poetry* texts contain, on average, the words with the highest surprisal, as opposed to *fiction*, with the lowest average surprisal score, followed by the texts composed based on *keywords*.

Overall, these statistics align with our expectations for the distinct text types.

**Table 6:** Descriptive statistics for each *decoding strategy*

		Text length	Word length	Zipf freq.	Flesch	Surp. GPT-2
Greedy Search	Mean	85.77	5.11	5.675	44.542	3.914
	Std	20.322	0.526	0.229	22.849	3.698
	Min	40.0	3.621	4.951	7.097	0.0
	Max	126.0	6.032	6.406	103.438	30.897
Beam Search	Mean	86.714	5.152	5.629	44.522	3.945
	Std	17.479	0.511	0.23	22.199	3.779
	Min	44.0	3.903	4.827	8.631	0.0
	Max	118.0	6.012	6.045	103.673	30.897
Sampling	Mean	85.817	5.208	5.629	41.517	4.116
	Std	20.824	0.524	0.233	22.381	3.836
	Min	34.0	3.853	4.889	5.949	0.0
	Max	127.0	6.66	6.117	108.073	30.897
Top-*K*	Mean	87.635	5.165	5.641	43.872	4.088
	Std	22.527	0.523	0.234	22.382	3.806
	Min	28.0	3.804	4.999	6.508	0.0
	Max	128.0	6.411	6.112	108.073	30.897
Top-*P*	Mean	86.706	5.163	5.64	41.218	4.002
	Std	21.166	0.511	0.223	23.321	3.727
	Min	25.0	3.795	4.952	4.783	0.0
	Max	130.0	6.33	6.191	105.246	30.897

Table 6 displays the lexical characteristics averaged over decoding strategies. There is little variability with respect to average text length, average word length, and Zipf frequency across decoding strategies (although beware that these metrics are computed after the post-processing of the generated texts, see Section “Post-processing of generated stimuli texts”). The greatest differences occur with regard to the Flesch reading ease: greedy search and beam search appear to produce slightly more difficult texts than random sampling and top-*p* sampling. Concerning surprisal, random sampling produces, on average, words with the highest surprisal value, although this stands somewhat in contrast to random sampling having the lowest Flesch reading ease score. Tables detailing these lexical characteristics for each model and each decoding strategy separately can be found in Appendix B.4.

**Fig. 1:**
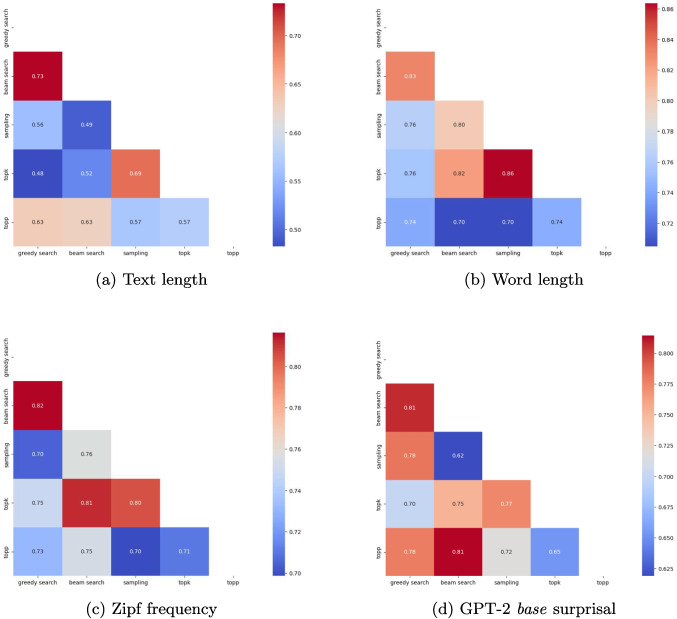
Correlations between texts produced by different decoding strategies for four different text metrics

We further compute the correlations between texts generated with the same decoding strategy (across all three LLMs) for the lexical characteristics text length, word length, Zipf frequency, and GPT-2 *base* surprisal, as depicted in Fig. 1. The greatest differences in correlation strength between the different decoding strategies occur when being computed for the text length feature, ranging from 0.48 (correlation between greedy search and top-*k* sampling) to 0.73 (beam search and greedy search). The least variability across correlation statistics occurs regarding surprisal extracted from the smaller GPT-2 *base* model, with the strongest correlations occurring for beam search and greedy search, and for beam search and top-*p* sampling. Since the decoding strategies can be grouped into two categories (likelihood maximization methods, which include greedy search and beam search; and stochastic methods, which include random sampling, top-*k* sampling, and top-*p* sampling), one might expect that those belonging to the same category displayed stronger correlations as opposed to across categories. However, for all lexical metrics except surprisal, top-*p* sampling seems to correlate less strongly with the other stochastic methods than with the likelihood maximization methods.

## Eye-tracking experiment

### Participants

In total, 119 native speakers of English with normal or corrected-to-normal vision participated in the experiment in Zurich, Switzerland. Out of these, we discarded the data of 12 participants due to insufficient data quality or bad calibration, which result in data of a total of 107 participants in the corpus. The participants come from various backgrounds and were recruited in a variety of ways: via the University of Zurich; Facebook groups such as *Expats in Switzerland*, *English-speaking jobs in Zurich*, and *Au-Pairs in Switzerland*; newsletters; the Democrats Abroad Switzerland; and the American Women’s Club Switzerland. Before commencing the experiment, they filled out a short questionnaire asking for their age, sex, amount of sleep, handedness, visual aids, possible second native languages, their English variant, astigmatism, myopia and hyperopia, and neurological disorders. An overview of selected characteristics is depicted in Table 7. All participant characteristics and information can be found in the file participant_info/participant_info.csv.

Participation requirements entailed being 18 years or older, having normal or corrected-to-normal vision, and being a native English speaker. There was no restriction pertaining to the variety of English; the distribution of English varieties spoken by the participants is displayed in Table 8. Participants were requested to not consume alcohol the night before or the day of the experiment. In case of wearing contact lenses, they were asked to wear their glasses, if available, and bring the contact lenses along. They were further asked not to wear eye makeup. Participants received a compensation of 30 CHF for an estimated 1 h of participation.

**Table 7:** Overview of the participants’ mean age and a selection of other statistics

Number of participants	Gender	Mean age ± sd	Mean hours of sleep ± (sd)	Visual aid$^{1}$	Myopia/hyperopia$^{2}$	Astigmatism
67	female	32.5 ± 12.0	7.3 ± 1.0	g: 36; s: 2	m: 31; h: 7	yes: 15
				n: 29	n: 29	no: 52
39	male	37.1 ± 15.7	7.0 ± 1.0	g: 21; s: 2	m: 15; h: 8	yes: 8
				n: 16	n: 16	no: 31
1	other	33	5	g: 1; n: -	m: 1; h: -	yes: 1
				n: -	n: -	no: -
107		34.1 ± 13.5	7.2 ± 1.0	g: 53; s: 4	m: 47; h: 15	yes: 24
				n: 50	n: 45	no: 83

$^{1}$ “g” $=$ glasses; “s” $=$ soft contact lenses; “n” $=$ no visual aid

$^{2}$ “m” $=$ myopic (short-sighted); “h” $=$ hyperopic (far-sighted), “n” $=$ neither

**Table 8:** Distribution of variants of English across participants

English variety	Number of speakers
American	45
British	33
Canadian	8
Australian	7
Indian	3
Irish	3
Scottish	3
New Zealand	2
Gibraltar	1
South African	1
Zimbabwe	1

### Experiment design

The 42 items in their 14 experimental conditions, where each condition consists of a combination of model and decoding strategy, were distributed across 14 lists in a Latin Square design to ensure that each participant sees each item only once, and each condition is presented to a participant the same number of times. Upon presentation, the order of the experimental items in each list was randomized. Each of the 42 trials consisted of four distinct parts: a header, the stimulus text, a comprehension question, and two rating questions. Each part was presented on its own screen. All text was presented in a mono-spaced font (Courier, font size 14) with double line spacing.

#### Header

The header is a sentence that introduces the subsequent text. Since the different texts are quite varied not only in content but also in form, the header’s purpose is to avoid the confusion of the reader and an initial skimming of the new text at hand. The headers are modeled as closely after the original model prompts as possible without revealing their original nature, i.e., that they were in fact prompts for a language model. We opted against displaying the prompts in their original form lest participants might discern that the texts were machine-generated. Furthermore, some prompts, such as the ones used for the summarization tasks, were too long to be presented on one single screen and would have anticipated the subsequent text’s content in a way that would have biased the readers’ eye movements.

#### Stimulus

The header is followed by the post-processed and validated generated text. Otherwise, the sequences are displayed as predicted by the models, including line breaks and white-space characters. However, for some texts, mostly the dialogues and some news articles, the models consistently predicted double newlines. In these cases, we removed one of the newline characters to ensure that the texts fit onto the presentation screen. This does not affect analyses with the data, however, as newlines are not areas of interest on their own, thus no fixations or reading measures are mapped onto them. From a psycholinguistic point of view, line breaks are important because words immediately preceding or following a line break exhibit a fixation pattern that would be different than if their position was further away from a line break. From a model perspective, these newline characters are also important, as tokens immediately following a newline character are conditioned on that newline character’s probability distribution and not on the distribution of the word preceding the newline. That being said, the texts as presented in the experiments exhibit more line breaks than originally predicted by the models, as they would not have fitted onto the screen otherwise. Assuming the psycholinguistic perspective, this does not change anything about the interpretation of fixations landing on tokens before or after newlines, though from the model perspective, especially when taking into account the transition scores of the generated tokens, there might be a slight bias, which was unavoidable unfortunately.

#### Comprehension question

Each text is followed by a multiple-choice comprehension question with four possible answers, out of which one answer is true. They serve the purpose of ensuring participants remain attentive and would allow to exclude data where people clicked through the texts and questions inattentively. The comprehension questions require inference from the text that was read, but with their main purpose being to maintain participants’ attention levels, they do not attempt to quantify their text comprehension.

#### Rating questions

After the comprehension question, participants answered two rating questions: the first inquired how subjectively difficult they deemed the text, and the second how engaging they found the text. Both questions were answered on a scale from 1 to 5.

### Procedure

The duration of one experiment session was around 60 min. Upon arrival, participants were asked to sign a declaration of consent and fill out the demographic questionnaire. Subsequently, the experimenter outlined the procedure of the experiment and provided them with instructions, which were both delivered verbally by the experimenter and again on the screen at the onset of the experiment: they were told to read the texts naturally, the way they would read a book or a newspaper, and that there were no time constraints; they should remain as still as possible and avoid asking questions or moving while reading the stimulus text. Upon calibration, the experiment started with a practice trial which would be discarded from the data. Before the appearance of both the header and the text on the presentation screen, participants were instructed to fixate on a fixation point at the position where the subsequent header and text would start. Drift correction was only applied if absolutely necessary. Participants could move from one screen to the next at their own pace by pressing the *Space* bar.

Responses to the questions were given with the numbers 1 to 4 (comprehension questions) or 1 to 5 (rating questions) on the keyboard. After reading the header or the text, participants were asked to focus on a blue sticker attached to the bottom right corner of screen before pressing the *Space* bar to proceed; this ensured that they did not fixate on the text at random between their finishing their reading process and pressing the key to continue. The experiment was divided into three sections; there was a 5-min break after 14 and after 28 experimental stimuli that allowed participants to quickly rest their eyes and get up. Re-calibration was performed after each break, and also in-between if necessary.

### Data acquisition and setup

The data acquisition took place in a controlled setting. Participants were placed in a sound-insulated and windowless room with controlled lighting. Participants put their head on a head-and-chin rest to limit head movement. They were instructed to remain immobile during the experiment, and were encouraged to rest their eyes and move their body during the breaks. They were seated at a height-adjustable table to warrant a constant eye-to-screen distance across participants. The eye-to-screen distance was 60 cm, with a 24-inch screen, and the distance from the eyes to the eye-tracker was 55 cm. The visual angle taken up by three characters is 1.05$^\circ$. Owing to the height-adjustable table, the head-and-chin rest did not need to be adjusted. We employed a resolution of 1280$\times$1024 pixels, which subsets the original screen size to one of 31 cm height and 43 cm width. The monitor refresh rate was 60 Hz. The experiment was implemented with Experiment Builder (SR Research Ltd., 2020).

Before commencing the experiment, the experimenter determined the participant’s dominant eye, which was then used for monocular eye-tracking. We tracked the eye position with a video-based infrared eye-tracker (EyeLink Portable Duo^16^), at a sampling rate of 2000 Hz. The eye-tracker was manually calibrated with a nine-point grid, the fixation points of that were displayed in random order. In a second step, the validation ensured that the error between two measurements at any point was less than 0.5$^\circ$.

## Eye-tracking data: Pre-processing

### Pre-processing of raw data

The data files written by the eye-tracker are non-human readable edf files. We converted them to asc with the edf2asc tool provided by SR Research. These asc files contain the data for one session, which not only include the recorded eye movement coordinates but also all metadata, such as button presses, drift corrections, re-calibrations, answers to the comprehension questions, and messages that indicate the onset and offset of individual trials and screens. These asc files are parsed and converted to csv to extract the relevant samples for each trial. The csv files consist of one line per sample, i.e., 2000 lines per second, which include timestamps as well as the *x*- and *y*-coordinates of where the tracked eye focussed. We tracked each participant’s dominant eye, so whether we tracked the left or the right one was participant-dependent, though all csv files bear the column names x and y for simplification purposes. The information on which eye was tracked for each participant can be found in participant_info/participant_info.csv.

**Fig. 2:**
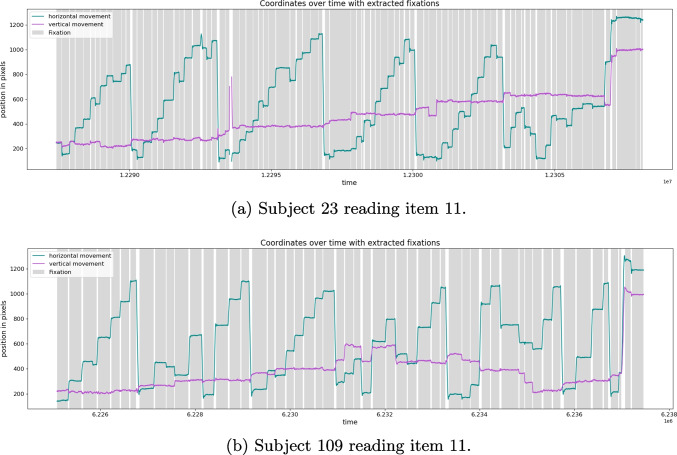
Horizontal and vertical eye movements during reading of one text, with *grey bars* indicating the fixations detected by the algorithm

### Fixation extraction

In order to group the raw data samples into fixations and saccades, we used the microsaccade detection algorithm introduced by Engbert and Kliegl (2003). We set the minimum fixation duration to 40 ms, the minimum saccade duration to 12 ms, and the maximum saccade velocity to 500 deg/s. This means that measures below the minimum and above the maximum thresholds were excluded during the fixation extraction. Saccades can be identified via their velocities, i.e., they are outliers in velocity space. Thus, the time series of coordinates of eye positions was first transformed into velocities as a weighted moving average over five data samples to suppress noise (Engbert, 2006), and then the detection threshold is computed as a multiple of the standard deviation of the velocity distribution. The detection thresholds are computed separately not only for each subject, but for each individual text, as proposed by Engbert et al. (2015), relative to the noise level, and they are multiplied by the threshold factor. Since the detection threshold is chosen with respect to the noise level of a single text, the algorithm is robust regarding different noise levels between texts as well as across participants. There are different methods to compute the threshold; we employed the one proposed by Engbert et al. (2015). Figure 2 shows the eye movements in terms of coordinates over time for two participants in two trials, where the green graph depicts the horizontal eye movement and the pink graph the vertical eye movement. The saccades detected by the algorithm are the white bars, and the grey bars are the fixations consequently. Although the two participants exhibit quite different reading behavior, the microsaccade detection algorithm efficiently and correctly groups and detects fixations and saccades for both.

**Fig. 3:**
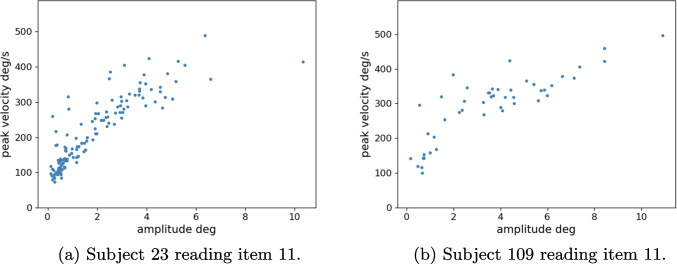
Saccade peak velocity in degrees of visual angle per second plotted over saccade amplitude in degrees

Since microsaccades, as well as saccades, are ballistic movements, i.e., rapid and forceful motions that leave little to no time for feedback mechanisms to make adjustments during the movement, they exhibit a fixed relation between peak velocity and amplitude. An inspection of this linear relationship between peak velocity and amplitude can thus serve as a kind of sanity check of the validity of the algorithm, i.e., whether or not the thresholds are correctly estimated and the saccades are accurately detected. Figure 3 depicts the peak velocities of saccades plotted over their amplitudes for two subjects reading two trials. The linear relationship is clearly discernible. Moreover, we also see that the algorithm sets an accurate upper bound regarding peak velocities by removing peak velocities greater than 500 degrees of visual angle per second. While it is indeed possible for human eyes to reach peak saccade velocities up to 1000 degrees per second, the upper bound for saccades during reading is around 500 degrees per second (Holmqvist et al., 2011); measurements with greater velocities are results of artifacts or blinks. Pertaining to saccade amplitude, there is no upper bound that can be systematically set. Figure 3 does indeed display outliers regarding the amplitude for both subjects where they moved their eyes entirely away from the text, but these outliers are removed in the manual fixation correction stage (see Section “Manual fixation correction”).

**Fig. 4:**
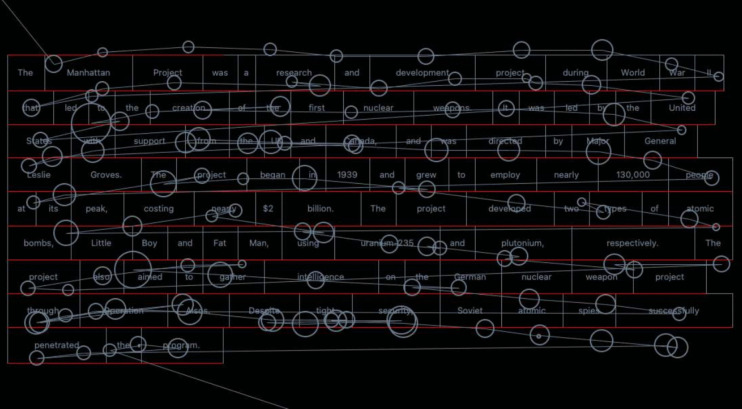
An example of vertical drift during the eye-tracking experiment. Subject 44 reading item43

### Manual fixation correction

A common issue that arises when conducting eye-tracking experiments is the so-called vertical drift, which means that there occurs a gradual displacement of the recorded coordinates over time (Carr et al., 2022). Phrased differently, coordinates are being recorded as being above or below the line that the participant is actually reading, which in turn results in fixations being placed above or below the lines on which they actually landed. This is particularly precarious when it comes to eye-tracking on the passage level, where participants read multi-line texts.

In such settings, vertical drift results in fixations being mapped to interest areas on the line above or below, which can distort the data. Vertical drift can occur even if the initial calibration was good and it is likely the result of deteriorating calibration over time or in the corners of the screen, or of subtle head movements, pupil dilations, teary eyes, and artifacts. Moreover, this vertical drift is usually not systematic across the entire screen and thus cannot be eliminated by drift correction. Figure 4 illustrates an example of a subject exhibiting vertical drift: there is an upward drift towards the middle of the screen, and a downward drift at the end of the line towards the bottom of the screen. These kinds of patterns cannot be remedied by drift correction. More examples of vertical drifts can be found in Appendix D.

In addition to vertical drift, there can also be instances of horizontal drift, which is unfortunately less recognizable than the vertical drift. However, since the areas of interest consist of words, we strongly assume that a possible horizontal measurement error will lead to only very few wrong fixation-to-word mappings, as it would have to be greater than the distance between the recorded fixation and the word boundary of its true area of interest.

Another problem leading to fixations being mapped to the wrong areas of interest is unrelated to the occurrence of vertical drift: participants might not immediately start reading once the text appears, but many first jump to the middle or the end of the text. Although these initial fixations might be considered part of a subject’s reading behavior, as they orient themselves within the text layout, they influence and distort the computation of reading measures. For instance, if a subject first jumps to the middle of the screen and then back to the beginning of the text, all words up to that first fixation in the middle would be attributed a first-pass reading time of zero.

Furthermore, some participants also moved their eyes outside the text during reading. These fixations are mapped to the closest area of interest, although they do not actually reflect true reading behavior. Additionally, since participants were instructed to move their eyes to a sticker located at the bottom right corner of the screen upon completing their reading, this area also contains several fixations. However, such eye movement behavior, which is not inherent to the actual reading of the text, is easily identifiable.

There exists a range of algorithms that implement an automatic vertical drift correction to amend the above-mentioned problems, such as the chain or the cluster algorithms (Schroeder, 2019), the compare algorithm (Sanches et al., 2015; Yamaya et al., 2017), the merge algorithm (Špakov et al., 2019), or the algorithms regress (Cohen, 2013) and segment (Abdulin & Komogortsev, 2015). However, these algorithms are all based on certain assumptions, *e.g.*, that the lines in a paragraph are read in chronological order, or they require to set threshold parameters that cannot be learned from the data. Moreover, they exhibit varying performance (Mercier et al., 2024). By providing manually corrected gold standard data together with the original uncorrected data, we enable other researchers to both evaluate and compare existing algorithms that correct for vertical drift, as well as develop new algorithms.

The manual fixation correction includes the mapping of all fixations exhibiting a vertical displacement to their nearest vertical areas of interest and removing the fixations that resulted from scanning the text before reading and jumping out of the text while reading. Although human eye movements in reading are interspersed with regressions and forward saccades, the underlying sequential nature of reading is still very well visible. While there might always be a chance of mapping errors, it is usually easy for a human annotator experienced with eye movements in reading to recognize the vertical drift and perform the fixation correction, a practice that is common in many eye-tracking labs.

### Computation of reading measures

From the fixation data, we computed a range of reading measures commonly used in reading research. The interest areas for the reading measures are equal to the ones of the fixation data; each reading measure is mapped to a white-space delimited word. This also entails that punctuation marks are not areas of interest on their own, but fixations landed on them are mapped to the preceding word or to the subsequent word in case of opening quotation marks or parentheses. The abbreviations and definitions of the computed reading measures are provided in Table 9.

**Table 9:** Definition and abbreviation of reading measures computed for EMTeC

Measure	Abbr.	Definition
*Continuous measures in ms*		
First-fixation duration	FFD	duration of the first fixation on a word if this word is fixated in first-pass reading, otherwise 0
First duration	FD	duration of the first fixation on a word (identical to FFD if not skipped in the first-pass)
First-pass reading time	FPRT	sum of the durations of all first-pass fixations on a word (0 if the word was skipped in the first-pass)
Single-fixation duration	SFD	duration of the only first-pass fixation on a word, 0 if the word was skipped or more than one fixations occurred in the first-pass (equals FFD in case of a single first-pass fixation)
First-reading time	FRT	sum of the duration of all fixations from first fixating the word (independent if the first fixations occurs in first-pass reading) until leaving the word for the first time (equals FPRT in case the word was fixated in the first-pass)
Total-fixation time	TFT	sum of all fixations on a word (FPRT+RRT)
Re-reading time	RRT	sum of the durations of all fixations on a word that do not belong to the first-pass (TFT-FPRT)
Inclusive regression-path duration	RPD_inc	sum of all fixation durations starting from the first first-pass fixation on a word until fixating a word to the right of this word (including all regressive fixations on previous words), 0 if the word was not fixated in the first-pass (RPD_exc+RBRT)
Exclusive regression-path duration	RPD_exc	sum of all fixation durations after initiating a first-pass regression from a word until fixating a word to the right of this word, without counting fixations on the word itself (RPD_inc-RBRT)
Right-bounded reading time	RBRT	sum of all fixation durations on a word until a word to the right of this word is fixated (RPD_inc-RPD_exc)
*Binary measures*		
Fixation	Fix	1 if the word was fixated, otherwise 0 (FPF or RR)
First-pass fixation	FPF	1 if the word was fixated in the first-pass, otherwise 0
First-pass regression	FPReg	1 if a regression was initiated in the first-pass reading of the word, otherwise 0 (RPD_exc)
Re-reading	RR	1 if the word was fixated after the first-pass reading, otherwise 0 (RRT)
*Discrete measures*		
Total fixation count	TFC	total number of fixations on that word
Incoming saccade length	SL_in	length of the saccade that leads to first fixation on a word in number of words; positive sign if the saccade is a progressive one, negative sign if it is a regression
Outgoing saccade length	SL_out	length of the first saccade that leaves the word in number of words; positive sign if the saccade is a progressive one, negative sign if it is a regression; 0 if the word is never fixated
Total count of incoming regressions	TRC_in	total number of regressive saccades initiated from this word
Total count of outgoing regressions	TRC_out	total number of regressive saccades landing on this word

## Analyses

### Descriptive statistics of the reading measures

We provide descriptive statistics of a selection of reading measures: the continuous measures *first-fixation duration* (FFD; duration of the first fixation on a word if it was fixated in first-pass reading), *first-pass reading time* (FPRT; sum of the durations of all first-pass fixations on a word), *total-fixation time* (TFT; sum of the durations of all fixations on a word), and *re-reading time* (RRT; sum of the durations of all fixations on a word that do not belong to the first-pass); and the binary measures *fixation* (Fix; whether or not a word was fixated) and *re-reading* (RR; whether or not a word was fixated after the first-pass reading).

Table 10 depicts statistics on the reading measures with respect to outputs of the individual models. We observe minimal variation both with respect to mean values as well as standard deviations between models, indicating that the reading behavior does not drastically change according to which model the respective texts were generated with. WizardLM seems to have slightly higher values across all metrics; for instance, words in texts generated by WizardLM display a higher re-reading time than words of texts originating from the other two models.

**Table 10:** Descriptive statistics of a selection of reading measures for each model. See Table 9 for a definition of the different reading measures$^{1}$

	Phi-2	Mistral	WizardLM
*Mean and standard deviation of continuous measures in ms*			
FFD	223.165 ± 101.362	222.166 ± 103.895	222.414 ± 100.985
FPRT	268.674 ± 154.69	265.261 ± 151.312	266.831 ± 151.84
TFT	326.477 ± 226.091	325.399 ± 230.474	327.748 ± 231.686
RRT	295.616 ± 213.118	300.596 ± 224.297	304.003 ± 229.13
*Mean proportions of the binary measures*			
Fix	0.694	0.694	0.695
RR	0.221	0.22	0.221

$^{1}$ For the statistics of FFD, FPRT, TFT, and RRT, words with a value of 0 (not fixated) were excluded

Statistics on the same reading measures pertaining to outputs generated with different decoding strategies are provided in Table 11. Again, there is little variability between the different decoding strategies regarding *first-fixation duration* and *first-pass reading time*, while there seems to be slightly greater variability concerning *total-fixation time* and *re-reading time*.

**Table 11:** Descriptive statistics of a selection of reading measures for each decoding strategy. See Table 9 for a definition of the different reading measures$^{1}$

	Greedy search	Beam search	
*Mean and standard deviation of continuous measures in ms*			
FFD	221.162 ± 101.095	223.534 ± 106.072	
FPRT	265.048 ± 152.407	266.682 ± 153.026	
TFT	323.098 ± 228.944	331.051 ± 239.462	
RRT	296.862 ± 222.448	309.743 ± 237.347	
*Mean proportions of the binary measures*			
Fix	0.693	0.692	
RR	0.22	0.223	
	Sampling	Top-*k*	Top-*p*
*Mean and standard deviation of continuous measures in ms*			
FFD	223.013 ± 102.286	223.297 ± 102.994	221.962 ± 99.678
FPRT	267.644 ± 153.084	267.573 ± 151.439	266.781 ± 152.463
TFT	329.343 ± 233.574	325.505 ± 224.761	325.131 ± 224.759
RRT	304.87 ± 228.032	296.272 ± 214.337	297.34 ± 216.7
*Mean proportions of the binary measures*			
Fix	0.697	0.696	0.693
RR	0.222	0.22	0.218

$^{1}$ For the statistics of FFD, FPRT, TFT, and RRT, words with a value of 0 (not fixated) were excluded

**Table 12:** Descriptive statistics of a selection of reading measures for each text type. See Table 9 for a definition of the different reading measures$^{1}$

	Non-fiction	Fiction	Poetry
*Mean and standard deviation of continuous measures in ms*			
FFD	220.79 ± 99.935	211.246 ± 90.626	242.242 ± 120.157
FPRT	262.891 ± 145.72	239.13 ± 122.998	296.138 ± 171.722
TFT	309.957 ± 208.142	272.512 ± 169.158	404.261 ± 284.231
RRT	285.469 ± 207.77	253.637 ± 165.551	349.679 ± 262.204
*Mean proportions of the binary measures*			
Fix	0.679	0.645	0.736
RR	0.189	0.17	0.333
	Summarization	Synopsis	Keywords
*Mean and standard deviation of continuous measures in ms*			
FFD	223.367 ± 100.731	223.991 ± 105.15	225.396 ± 105.051
FPRT	270.289 ± 154.528	273.739 ± 162.574	275.374 ± 162.256
TFT	341.677 ± 239.886	342.11 ± 246.012	338.416 ± 244.337
RRT	308.318 ± 226.802	310.199 ± 230.016	308.105 ± 236.737
*Mean proportions of the binary measures*			
Fix	0.724	0.718	0.696
RR	0.257	0.24	0.222

$^{1}$For the statistics of FFD, FPRT, TFT, and RRT, words with a value of 0 (not fixated) were excluded.

More distinct differences emerge when examining the reading measures with respect to the text types, as displayed in Table 12. While *fiction* has the lowest *first-fixation duration* and *first-pass reading time*, *poetry* has by far the longest *first-fixation durations* and *first-pass reading times*. This is also reflected in *total-fixation time*: the words in the poems are, on average, fixated for the longest amount of time, while those in fictional texts are fixated on for the shortest duration. This higher value of TFT in poems is probably accounted for by a longer re-reading time compared to the other text types.

**Fig. 5:**
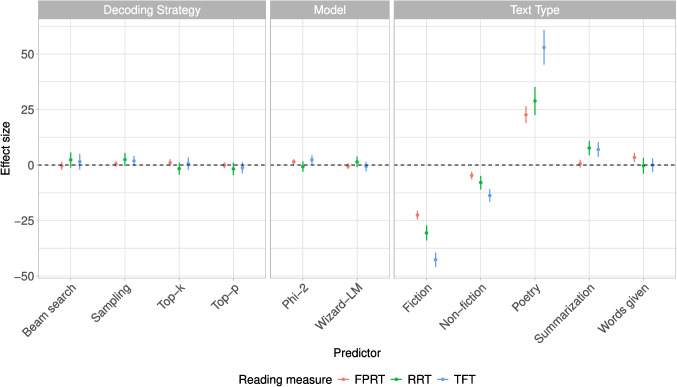
Effect sizes in milliseconds (mean and 95% credible interval) of sum-contrast-coded predictors *decoding strategy*, *model*, and *task* on the different reading measures, first-pass reading time (FPRT), re-reading time (RRT), and total fixation time (TFT)

### The effects of model, decoding strategy, and text type on reading times

To corroborate the trends indicated by the descriptive statistics in Section “Descriptive statistics of the reading measures”, i.e., that words belonging to certain text types or to texts generated with certain decoding strategies or by certain models are fixated for a longer time overall, we perform a Bayesian analysis that investigates whether these three factors (i.e., model, decoding strategy, and text type) have an effect on the reading time on that word. To that end, we fit linear-mixed models $\mathcal {M}_{\beta }: \mathbb {R}^N \rightarrow \mathbb {R}$ that map from $N$ predictors to a log-transformed reading measure $y_{ij}$ obtained from subject $j$ on word $i$, and that is parametrized by $\varvec{\beta }$. More specifically, we formalize our model as

11 $$\begin{aligned} \mathcal {M}_{\beta }: y_{ij} \sim \beta _0 + \beta _{0j} + (\beta _1 + b_{1j})\,dec_i + (\beta _2 + b_{2j})\,model_i + (\beta _3 + b_{3j})\,tt_i, \end{aligned}$$

where $\beta _0$ is the intercept; $\beta _{0j}$ is the by-subject random intercept of subject $j$; $dec_i$, $model_i$, and $tt_i$ are the predictor variables decoding strategy, model, and text type associated with word $i$; $\beta _{1 \dots 3}$ are the coefficients of the predictor variables; and $b_{1 \dots 3}$ are random by-subject slopes for those predictors. The predictor variables are all sum-contrast coded. As response variable $y_{ij}$, we use the continuous reading measures *first-pass reading time* (FPRT) as a representative for early measures of language processing; *total fixation time* (TFT) as global measure; and *re-reading time* (RRT) as a representative for late measures. To fit the model, we use the brms-package (Bürkner, 2017) and run each model for 3000 iterations, including 1000 warm-up iterations. We use standard uninformed priors, reported in Appendix C. We present posterior distributions (mean and 95% credible intervals) for all coefficients, back-transformed to milli-seconds in Fig. 5.

**Fig. 6:**
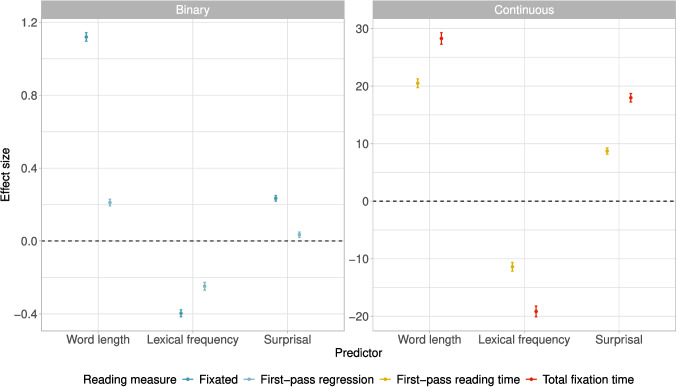
Posterior distributions (mean and 95% credible interval) over the word length, lexical frequency, and surprisal coefficients, estimated via the Bayesian hierarchical model presented in Eq. 12. The effect sizes (*y*-axis) represent milliseconds for the continuous reading measures and log-odds for the binary reading measures

Overall, the figure shows that, relatively speaking, the largest differences between conditions can be observed for the different text types (right panel). With the text type of *article synopsis* subsumed in the grand mean comparison, the coefficients indicate that the words in texts belonging both to the text types *non-fiction* and *fiction* are fixated for shorter-than-average, reflected in all three reading measures FPRT, RRT, and TFT. *Poetry*, on the other hand, is a text type whose words are fixated for longer overall, again across all reading measures. For *summarization* and *keyword texts*, the picture is more varied and depends on whether we are considering early, late, or global processes. More specifically, words in *keyword texts* have a higher-than-average FPRT, while words in *summarization* texts have a higher-than-average TFT and RRT.

The effects for both the decoding strategy as well as the model with which the texts were generated are much smaller and do not provide a conclusive picture. However, we observe a weak trend for higher re-reading times and total-fixation times in texts generated with ancestral sampling. Moreover, the results suggest a weak trend for higher total-fixation times on words in texts generated with Phi-2 than those generated with the other models.

### Bayesian psycholinguistic analysis

In addition to the analyses above, we also assess the effects of various word-level psycholinguistic features on four reading measures: *first-pass reading time* (FPRT; sum of the durations of all first-pass fixations on a word), *total fixation time* (TFT; sum of the durations of all fixations on a word), *fixation proportion* (Fix; whether or not a word was fixated), and *first-pass regressions* (FPReg; whether a regression was initiated in the first-pass reading of a word). To do so, we deploy hierarchical linear-mixed models with reading measures $y_{ij}$ obtained for word $j\in \{1\dots J\}$, where *J* is the total number of words across all stimulus texts^17^ read by participant *i* as response variables, and normalized (z-score transformed) predictors word length $l_j$ (number of characters including punctuation), Zipf frequency $f_j$, lexicalized surprisal (GPT-2 *base*) $s_j$, and last in line $t_j$ (binary: 1 if the word included a line-break), formalized in Equation 12

12 $$\begin{aligned} y_{ij} = L(\beta _0 + \beta _{0i} + \beta _{1}{l}_{j} + \beta _{2}{f}_{j} + \beta _{3}{s}_{j} +\beta _{4} t_j )\quad , \end{aligned}$$

where $\beta _0$ denotes the global, and $\beta _{0i}$ the random intercept for subject *i*. $L(\cdot )$ denotes the linking function, in the case of the binary measures (*first-pass regression*, *fixation proportion*), $L(z)=\ln \frac{z}{1-z}$, with $y_{ij}$ following a Bernoulli distribution; and in case of the continuous measures (*first-pass reading time*, *total fixation time*) $L(z)=\log z$, with $y_{ij}$ following a log-normal distribution. We use the same configuration (priors, number of iterations) as in the lexical feature analysis presented in Section “Bayesian psycholinguistic analysis”.

We present posterior distributions (mean and 95% credible intervals) for the word length, lexical frequency, and surprisal coefficients in Fig. 6. For continuous variables, the effect sizes are in milliseconds, for the binary variables in log-odds. We see that all predictors are in line with their effect on processing effort: longer words induce longer reading times, are more likely to be fixated, and are more likely to lead to a successive regression. The same pattern can be observed for high-surprisal words as well as for low-frequency words.

### Response accuracy

To investigate whether participants’ comprehension accuracy in answering the comprehension question differed with respect to whether the text was generated by a certain model, with a certain decoding strategy, or belongs to a specific text type, we aggregate participants’ accuracies across all texts belonging to either of these three factors.

**Table 13:** Mean response accuracy across texts generated by a specific LLM, with a specific decoding strategy, or belonging to a certain text type

Factor	Conditions	Accuracy
Model	Phi-2	0.84
	Mistral	0.83
	WizardLM	0.86
Decoding strategy	Greedy search	0.89
	Beam search	0.87
	Sampling	0.79
	Top-$k$	0.83
	Top-$p$	0.84
Task	Non-fiction	0.89
	Fiction	0.83
	Poetry	0.73
	Summarization	0.85
	Article synopsis	0.84
	Keyword text	0.86

As is depicted in Table 13, the response accuracy is comparable across different LLMs, across different decoding strategies, and across different text types, although there are minor variations, such as the decoding strategy *sampling* with the lowest response accuracy of 0.79, or the text type *poetry* with the lowest accuracy of 0.73. Overall, however, participants seem to have been consistent in correctly answering the comprehension questions regardless of model, decoding strategy, and text type.

While we did find in Section “The effects of model, decoding strategy, and text type on reading times” that words belonging to certain text types are fixated for longer or shorter than average, the differences in reading behavior are not reflected in participants’ response accuracies. This finding can serve as a sanity check, as the comprehension questions have been primarily included in the experiment to ensure that participants remain attentive during reading. Moreover, since the purpose of the comprehension questions was merely for participants to maintain their attention level, participants’ response accuracies do not allow for drawing conclusions about the difficulty of the texts.

## Accessing the data

Since EMTeC consists of different types of data, we have opted for distributing it across different channels. All code implementations are available via this GitHub repository^18^. This repository can be cloned and the eye-tracking data files can be automatically downloaded using a Python script. Alternatively, the eye-tracking data can also be downloaded via this Open Science Framework (OSF) repository^19^. The transition scores, attention scores, and hidden states are very large tensors and require a large amount of disk space to be stored.^20^ We make these available via this Harvard Dataverse Dataset^21^.

## On the use cases of EMTeC

Below we present a (non-exhaustive) list of the use cases of EMTeC. There are different axes to the EMTeC data that can all be leveraged for a variety of intents and purposes: the eye movement data at different stages of pre-processing; the uncorrected and corrected data versions; the different text types; and the machine-generated nature of the texts and the model internals.

The **eye movement data at different pre-processing stages** includes the raw data consisting of coordinate-timestamp samples, the fixation sequence data, and the reading measures. As suggested by Jakobi et al. (2024), we share the data at all three stages of pre-processing to allow for a broad range of use cases.

The **raw data** can be used for

- the development of pre-processing and data filtering methods such as blink detection algorithms (Królak & Strumiłło, 2012; Morris et al., 2002);
- the development of new gaze event detection algorithms, such as saccade and fixation detection algorithms (Nyström & Holmqvist, 2010; Olsson, 2007; Salvucci & Goldberg, 2000; Sauter et al., 1991; Smeets & Hooge, 2003);
- the training and evaluation of generative models of human eye movements that generate raw data samples (Prasse et al., 2023, 2024);
- and research on vision and oculomotor control, such as the investigation of oculomotor micro-movements (Engbert, 2006; Martinez-Conde et al., 2006, 2009) or the velocity and acceleration profiles of saccades (Jäntti et al., 2011; Bachurina & Arsalidou, 2022).

Raw data provides researchers with the highest flexibility when using the data, allowing them for applying pre-processing algorithms different from the ones used in this paper.

The **fixation sequence data** can be used for

- the training and evaluation of generative models of eye movements in reading that simulate scanpaths on textual stimuli (Bolliger et al., 2023; Deng et al., 2023; Hahn & Keller, 2016; Nilsson & Nivre, 2009, 2011; Wang et al., 2019);
- the psycholinguistic analysis of scanpaths to gain insights into the cognitive processes involved in reading (von der Malsburg & Vasishth, 2013; von der Malsburg & Vasishth, 2011; von der Malsburg et al., 2012);
- the enhancement of language models with scanpaths (gaze-augmented language models) (Deng et al., 2023; Khurana et al., 2023; Yang & Hollenstein, 2023; Deng et al., 2024);
- and the development of metrics that quantify the differences and similarities between scanpaths (Cristino et al., 2010; Jarodzka et al., 2010; Mathôt et al., 2012; Shepherd et al., 2010; von der Malsburg & Vasishth, 2011).

The **reading measures** together with the linguistic annotations of the stimulus texts can be used for

- statistical analyses to evaluate and develop psycholinguistic theories of eye movements in reading. This includes, but is not limited to, investigations of phenomena at the lexical level such as word-length and lexical frequency effects, or the syntactic level, such as surprisal effects (Demberg & Keller, 2008; Hoover et al., 2023; Shain, 2021; Pimentel et al., 2023), and locality or anti-locality effects (Bartek et al., 2011; Demberg & Keller, 2008);^22^
- investigations of the predictive power of surprisal (Oh & Schuler, 2023b, a);
- the cognitive enhancement of language models (i.e., improving the performance of LMs on downstream tasks by integrating data such as eye movements in reading, that is, behavioral data reflecting cognitive processes) (Cheri et al., 2016; Deng et al., 2023; Hollenstein & Zhang, 2019; Hollenstein et al., 2020, 2021b; Long et al., 2017; Malmaud et al., 2020; Mishra et al., 2017a; Sood et al., 2020; Takmaz et al., 2020; Tiwari et al., 2023; Wang et al., 2024; Yang & Hollenstein, 2023);
- research on the cognitive interpretability and plausibility of language models (Beinborn & Hollenstein, 2023; Bensemann et al., 2022; Eberle et al., 2022; Keller, 2010; Hollenstein et al., 2021a; Hollenstein & Beinborn, 2021; Hollenstein et al., 2022b; Merkx & Frank, 2021; Sood et al., 2020);
- and the investigation of text difficulty and whether it is reflected in the reading behavior, together with the provided text difficulty metrics and participants’ responses to comprehension question and subjective text difficulty.

The **uncorrected and corrected versions of the data** can be used for

- the evaluation of rule-based algorithms that correct vertical drift in eye-tracking data (Abdulin & Komogortsev, 2015; Sanches et al., 2015; Schroeder, 2019; Špakov et al., 2019; Yamaya et al., 2017)
- and both the training and evaluation of machine learning-based algorithms correcting vertical drift in eye-tracking data (Cohen, 2013).

The information on the different **text types** can be used for

- the inference of text types from eye movement data;
- and research on reading behavior as a function of the type of text that is being read (poetic, argumentative, etc.), and, relatedly, investigations of the impact of different text structures and layouts on reading behavior, such as poems, dialogues, and news articles.

And last, the nature of the texts as being **machine-generated** by **different language models**, involving the information on the **decoding strategies** used by the LMs as well as the provided **transition scores, attention scores, and hidden states** can be used for

- the investigation of the cognitive alignment of the output of specific LMs with humans via transition scores and attention scores;
- the investigation of the cognitive alignment of the output of specific decoding strategies with humans via transition scores and attention scores;
- the investigation of reading behavior on machine-generated texts, furthering the automated detection of machine-generated texts;
- the evaluation of the quality of machine-generated text with eye movements;
- the leveraging of eye movements to train a reward model for reinforcement learning from human feedback (RLHF);
- the investigation of the predictive power of surprisal estimated directly for texts that were machine-generated;
- and the investigation of the predictive power of surprisal when computed directly from the transition scores.

## Conclusion

We presented EMTeC, the **E**ye Movements on **M**achine-Generated **Te**xts **C**orpus, a naturalistic English eye-tracking-while-reading corpus. EMTeC is the first eye-tracking corpus whose experimental stimuli were generated with different large language models utilizing five different decoding strategies. The language models are of different size and belong to different model families, and the texts can be categorized into six different types, including fiction, poetry, and summarization. The corpus further entails the language model internals during generation, i.e., the transition scores, the attention scores, and the hidden states. Moreover, the stimuli have been linguistically annotated with a variety of features, both on text- as well as on word-level. The eye movement data is made available at all stages of pre-processing, that is, the raw coordinate data, the fixation sequences, and the reading measures. To establish transparency and ensure reproducibility, we also release the code accompanying the corpus, i.e., the scripts used for the generation of the stimuli, for the linguistic annotation, for the pre-processing of the eye movement data, and for the psycholinguistic analysis. We also provide both the uncorrected and corrected versions of the fixation sequences, along with the reading measures computed from them. This – i.e., the data at all pre-processing stages, the code, and the corrected and uncorrected data version – provides the user with maximal flexibility when choosing the data type that best suits their purposes and research questions, as well as allowing for the application of pre-processing algorithms different from the ones we have employed.

Based on the features described above and the nature of the corpus, we anticipate EMTeC to be utilized for a variety of use cases that range from the development of pre-processing and gaze event detection algorithms to research on vision and oculomotor control, the training and evaluation of generative models of eye movements in reading, the development of scanpath similarity metrics, the cognitive enhancement and the cognitive interpretability of language models, psycholinguistic analyses of the scanpaths and the reading measures as well as the development and evaluation of psycholinguistic theories, the investigation of the predictive power of surprisal, the investigation of text difficulty, the training and evaluation of drift correction models, and research on reading behavior as a function of the type of text that is being read.

This list of use cases is non-exhaustive, and we envision EMTeC to be used for more intents and purposes that go beyond NLP, psycholinguistics, and reading research. EMTeC falls into a canon of a new standard of publishing eye-tracking data at multiple stages of its development and reporting its quality, and it aims to further the collection and publication of eye-tracking data in a similar manner, complementing existing data.

## Figures and Tables

**Fig. 7:**
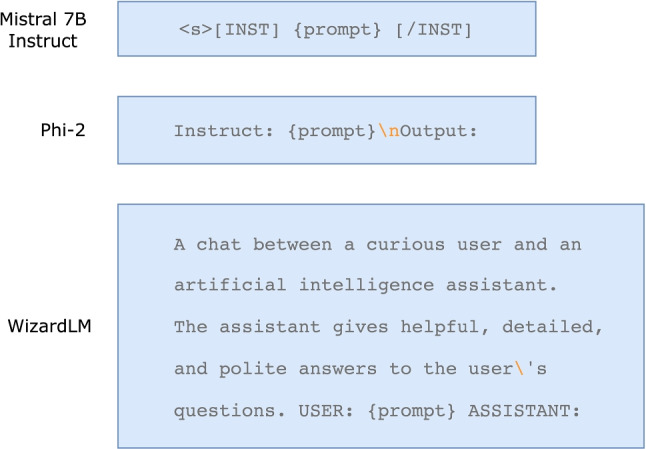
Prompt templates used for Mistral 7B Instruct, Phi-2, and WizardLM. The *curly brackets* {prompt} indicate the placeholder for the actual prompt

**Fig. 8:**
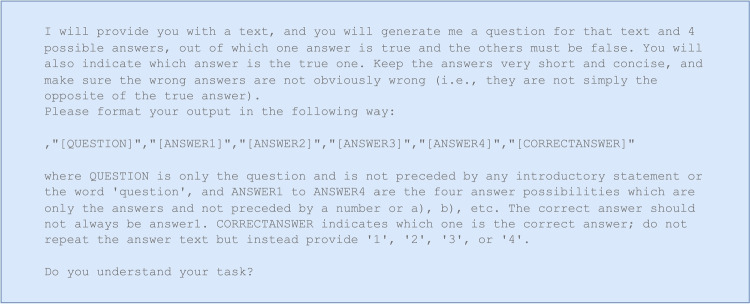
Prompt template used for ChatGPT to generate the comprehension questions

**Fig. 9:**
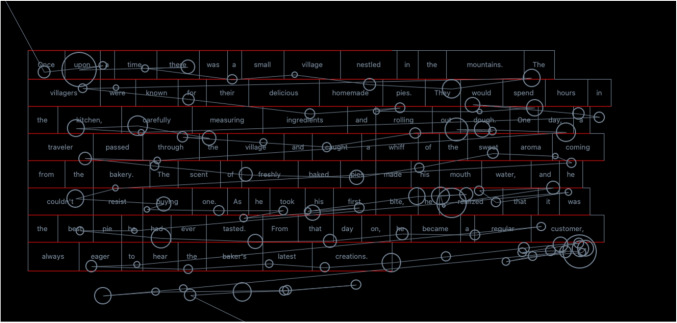
An example of vertical drift during the eye-tracking experiment. Subject 28 reading item06

**Fig. 10:**
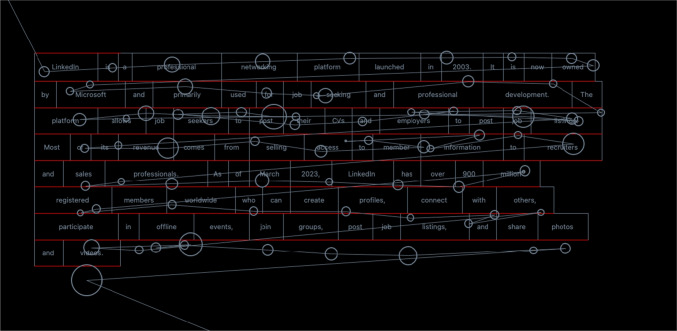
An example of vertical drift during the eye-tracking experiment. Subject 28 reading item35

**Fig. 11:**
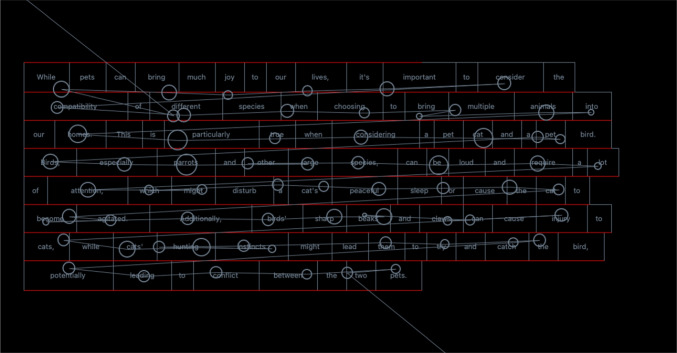
An example of vertical drift during the eye-tracking experiment. Subject 42 reading item01

**Fig. 12:**
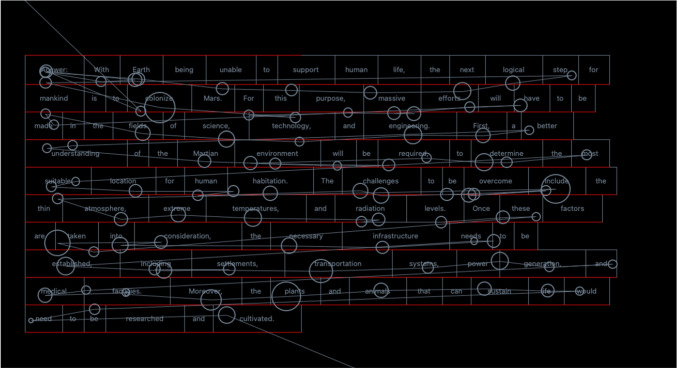
An example of vertical drift during the eye-tracking experiment. Subject 42 reading item72

**Fig. 13:**
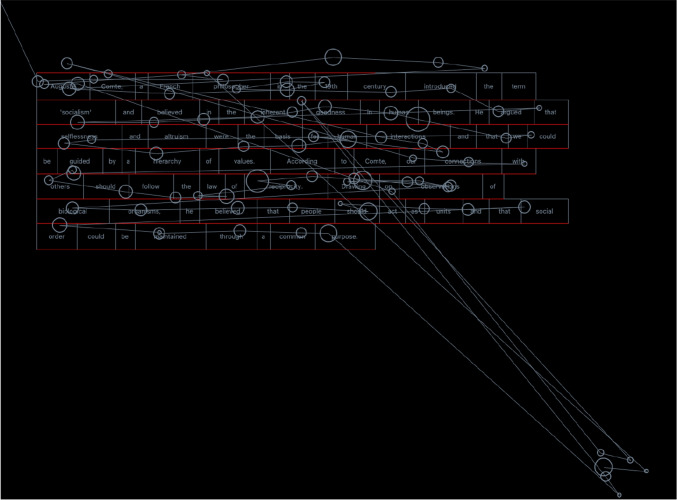
An example of vertical drift during the eye-tracking experiment, including jumps out of the text to the blue sticker and back into the text. Subject 49 reading item51

**Fig. 14:**
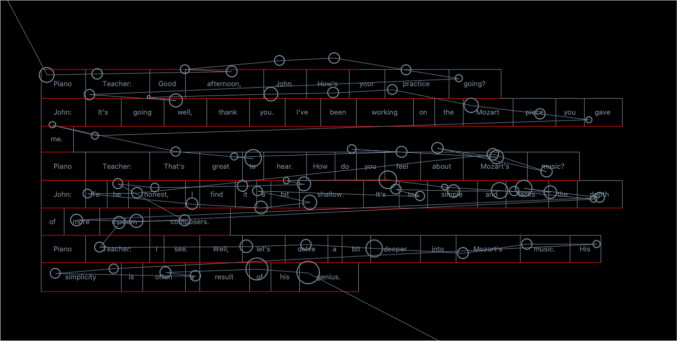
An example of vertical drift during the eye-tracking experiment. Subject 49 reading item80

**Fig. 15:**
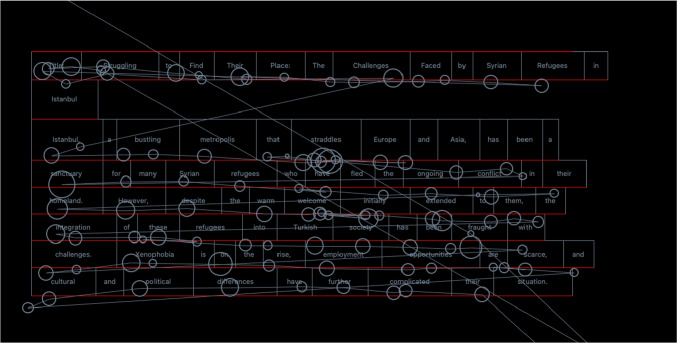
An example of vertical drift during the eye-tracking experiment. Subject 50 reading item46

**Fig. 16:**
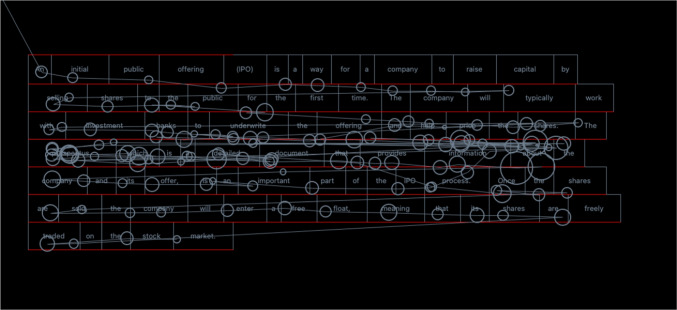
An example of vertical drift during the eye-tracking experiment. Subject 50 reading item53

**Fig. 17:**
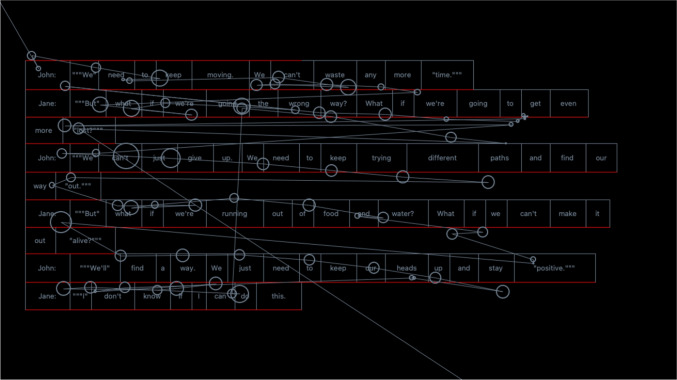
An example of vertical drift during the eye-tracking experiment. Subject 67 reading item11

**Fig. 18:**
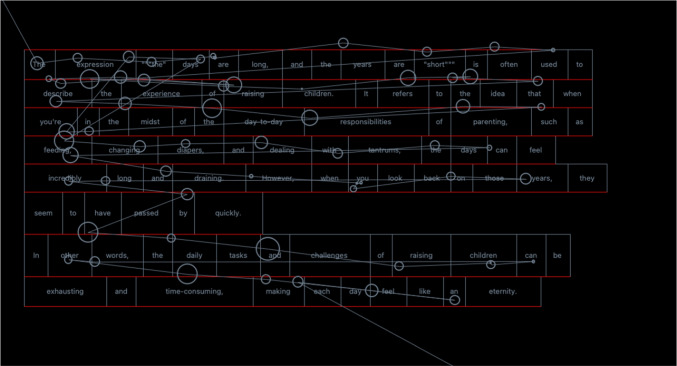
An example of vertical drift during the eye-tracking experiment. Subject 67 reading item14

**Fig. 19:**
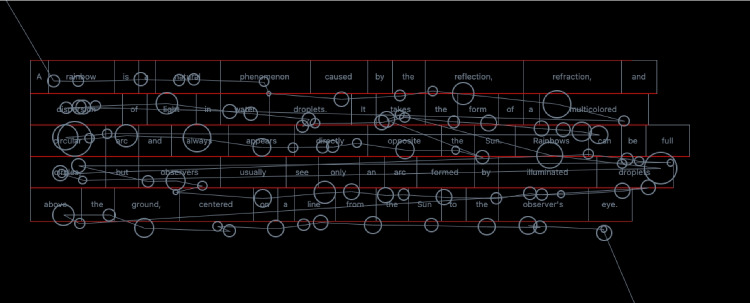
An example of vertical drift during the eye-tracking experiment. Subject 81 reading item40

**Fig. 20:**
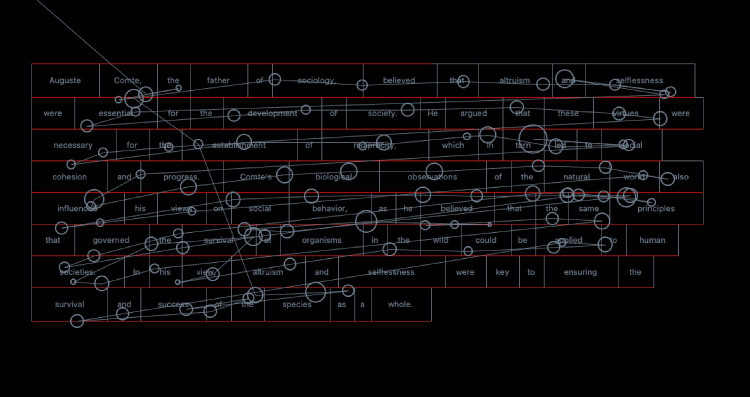
An example of vertical drift during the eye-tracking experiment. Subject 83 reading item51

## Data Availability

The data is available via this GitHub repository(https://github.com/DiLi-Lab/EMTeC/). Alternatively, the eye-tracking data can be downloaded via this Open Science Framework (OSF) repository (https://osf.io/ajqze/), and the model internals via this Harvard Dataverse repository(https://dataverse.harvard.edu/dataset.xhtml?persistentId=doi:10.7910/DVN/GCU0W8).

## Appendix A: Single-sentence corpora of eye movements in reading

Eye-movement corpora containing data of subjects reading single sentences can be categorized into those corpora that comprise *partially constructed stimuli* and those comprising *naturalistic stimuli*. An overview of the corpora mentioned below is provided in Table 14.

Partially constructed stimuli typically concentrate on linguistic constructions that might not occur frequently enough in naturalistic language examples and are thus authored for the purpose of studying different phenomena. For instance, the Potsdam Sentence Corpus (Kliegl et al., 2004) consists of 144 German sentences that entail a variety of linguistic structures, read by 33 younger 32 older adults. Other single-sentence corpora are TURead (Acartürk et al., 2023) with 196 participants reading Turkish stimuli; The Potsdam-Allahabad Hindi Eyetracking Corpus (Husain et al., 2014), containing 30 participants reading 153 sentences in Hindi or Urdu; a Chinese dataset with the purpose of studying word length effects, comprising 30 participants reading 90 Chinese sentences (Zang et al., 2018); and the Russian Sentence Corpus (Laurinavichyute et al., 2018), with 96 participants reading 144 sentences.

Naturalistic single-sentence corpora, on the other hand, involve the presentation of sentences that occur in natural language. The vast majority of these corpora are based on English texts. The Corpus of Eye Movements in L1 and L2 English Reading (CELER) (Berzak et al., 2022) is the largest corpus in terms of number of participants, with a total of 365 subjects (roughly 80% of which are non-native speakers) reading 156 English sentences from the Wall Street Journal. As another example, The Zurich Cognitive Language Processing Corpora, ZuCo (Hollenstein et al., 2018) and ZuCo 2 (Hollenstein et al., 2020), comprise 12 and 18 native English speakers, respectively, who were concomitantly tracked with EEG. Other corpora include the UCL corpus (Frank et al., 2013) with 43 participants reading sentences from English novels; RaCCooNS (Frank & Aumeistere, 2023) with 37 subjects reading 200 narrative sentences; the CFILT sarcasm dataset (Mishra et al., 2016), entailing seven non-native English speakers reading sarcastic and non-sarcastic sentences; and the CFILT sentiment complexity dataset (Joshi et al., 2014), where five participants rated the sentiment of over 1000 English sentences. Chinese single-sentence corpora involve the Hong Kong Corpus of Chinese Sentence and Passage Reading (Wu & Kit, 2023), including 96 participants that read 300 sentences and seven multi-line passages; the Beijing Sentence Corpus (Pan et al., 2021), with 60 participants reading 150 sentences from People’s Daily; and Zhang et al. (2022) tracked 1718 subjects reading over 7500 Chinese sentences.

**Table 14:** Single-sentence eye movement corpora categorized by stimulus type

Corpus Name	Language(s)	Details
*Partially Constructed Stimuli*		
Potsdam Sentence Corpus (Kliegl et al., 2004)	German	144 sentences, 65 participants
TURead (Acartürk et al., 2023)	Turkish	192 sentences, 196 participants
Potsdam-Allahabad Hindi Eyetracking Corpus (Husain et al., 2014)	Hindi, Urdu	153 sentences, 30 participants
Chinese Word Length Study (Zang et al., 2018)	Chinese	90 sentences, 30 participants
Russian Sentence Corpus (Laurinavichyute et al., 2018)	Russian	144 sentences, 96 participants
*Naturalistic Stimuli*		
CELER (Berzak et al., 2022)	English	156 sentences, 365 participants (80% non-native)
ZuCo (Hollenstein et al., 2018)	English	1059 sentences, 12 participants participants, tracked with EEG
ZuCo 2 (Hollenstein et al., 2020)	English	739 sentences, 18 participants, tracked with EEG
UCL Corpus (Frank et al., 2013)	English	361 sentences from novels, 43 participants
RaCCooNS (Frank & Aumeistere, 2023)	English	200 narrative sentences, 37 participants
CFILT Sarcasm Dataset (Mishra et al., 2016)	English	1000 sarcastic/non-sarcastic sentences, 7 non-native participants
CFILT Sentiment Complexity Dataset (Joshi et al., 2014)	English	1059 sentences, 5 participants
Hong Kong Corpus (Wu & Kit, 2023)	Chinese	300 sentences, 7 passages, 96 participants
Beijing Sentence Corpus (Pan et al., 2021)	Chinese	150 sentences, 60 participants
Chinese Sentence Corpus (Zhang et al., 2022)	Chinese	7500 sentences, 1718 participants

## Appendix B: Stimuli

### B.1 Model choice

We opted for Phi-2 (Javaheripi et al., 2023), Mistral 7B Instruct (Jiang et al., 2023) and WizardLM (Xu et al., 2023) as they yielded the best output from an eye-tracking experiment point-of-view. Since the participants were not informed about the fact that all texts were machine-generated, exclusion criteria entailed outputs that were frequently self-referential (e.g., *As an AI model, ...*), repetition of the prompt in the output, and repetitions within the output. We not only wanted to have eye movements during natural reading, which would be disturbed by sentences being repeated, but we also did not want participants to know about the nature of the texts, lest they might be primed to search for AI cues.

We experimented with other models: GPT-2 (Radford et al., 2019) (the deterministic outputs, i.e., greedy search and beam search, were not suitable as they contained a lot of repetition; Falcon-40B Instruct (Almazrouei et al., 2023) (non-sensical and self-referential output); Llama-2-70B Instruct (Touvron et al., 2023) (computationally not feasibly); XGen (Nijkamp et al., 2023) (the output would have been suitable from a quality perspective, but the model did not predict white-space characters, so truncating and pooling to word level would have been difficult); and MPT-30B-Instruct (Team, 2023)(irregularities in the prediction of white-space characters that made truncation and pooling difficult).

### B.4 Descriptive statistics of the stimuli

**Table 15:** Descriptive statistics of the average text length, word length, Zipf frequency, Flesch reading ease, and GPT-2 surprisal for each decoding strategy used by Mistral

		Text length	Word length	Zipf freq.	Flesch	Surp. GPT-2
Greedy search	Mean	89.881	5.11	5.678	49.261	3.891
	Std	19.042	0.509	0.247	19.32	3.653
	Min	44.0	3.621	5.153	17.836	0.0
	Max	118.0	5.899	6.406	99.814	30.897
Beam search	Mean	90.548	5.137	5.641	47.689	3.91
	Std	19.267	0.473	0.237	18.688	3.748
	Min	44.0	4.086	4.943	16.493	0.0
	Max	114.0	5.947	6.045	100.731	30.897
Sampling	Mean	88.595	5.176	5.634	46.114	4.031
	Std	18.456	0.461	0.222	17.547	3.786
	Min	37.0	4.362	4.999	5.949	0.0
	Max	117.0	6.66	6.112	83.854	30.897
Top-*K*	Mean	92.0	5.16	5.638	47.107	3.963
	Std	19.44	0.432	0.235	17.733	3.707
	Min	37.0	4.362	4.999	6.508	0.0
	Max	124.0	5.949	6.112	83.854	30.897
Top-*P*	Mean	88.952	5.192	5.622	43.891	3.881
	Std	20.889	0.447	0.236	17.556	3.671
	Min	37.0	4.141	5.146	6.194	0.0
	Max	119.0	6.33	6.191	89.08	30.897

**Table 16:** Descriptive statistics of the average text length, word length, Zipf frequency, Flesch reading ease, and GPT-2 surprisal for each decoding strategy used by Phi-2

		Text length	Word length	Zipf freq.	Flesch	Surp. GPT-2
Greedy search	Mean	82.595	5.101	5.726	47.348	3.813
	Std	22.568	0.547	0.18	21.134	3.64
	Min	41.0	3.901	5.366	11.623	0.0
	Max	126.0	6.032	6.189	103.438	30.897
Sampling	Mean	83.024	5.211	5.648	44.002	4.1
	Std	27.542	0.603	0.251	22.009	3.8
	Min	34.0	3.853	4.889	16.549	0.0
	Max	127.0	6.411	6.094	105.304	30.897
Top-*K*	Mean	85.714	5.114	5.687	47.396	4.052
	Std	28.905	0.59	0.213	22.216	3.759
	Min	30.0	3.804	5.128	18.189	0.0
	Max	128.0	6.411	6.083	105.304	30.897
Top-*P*	Mean	85.976	5.098	5.684	46.856	4.035
	Std	26.321	0.558	0.217	21.865	3.703
	Min	25.0	3.795	4.952	14.999	0.0
	Max	130.0	6.028	6.086	105.246	29.314

**Table 17:** Descriptive statistics of the average text length, word length, Zipf frequency, Flesch reading ease, and GPT-2 surprisal for each decoding strategy used by WizardLM

		Text length	Word length	Zipf freq.	Flesch	Surp. Gpt-2
Greedy search	Mean	84.833	5.118	5.623	46.237	4.037
	Std	18.939	0.535	0.246	20.407	3.798
	Min	40.0	4.184	4.951	7.097	0.0
	Max	118.0	6.012	6.041	94.045	30.897
Beam search	Mean	82.881	5.167	5.618	44.762	3.984
	Std	14.737	0.552	0.225	22.727	3.812
	Min	49.0	3.903	4.827	8.631	0.0
	Max	118.0	6.012	6.035	103.673	24.775
Sampling	Mean	85.833	5.239	5.606	44.131	4.22
	Std	14.385	0.508	0.228	20.123	3.919
	Min	48.0	4.077	5.015	9.981	0.0
	Max	111.0	6.303	6.117	108.073	29.314
Top-*K*	Mean	85.19	5.221	5.598	45.118	4.262
	Std	17.444	0.542	0.248	20.753	3.95
	Min	28.0	4.015	5.103	10.783	0.0
	Max	114.0	6.405	5.979	108.073	30.897
Top-*P*	Mean	85.19	5.199	5.613	45.188	4.097
	Std	15.121	0.529	0.212	21.432	3.806
	Min	44.0	4.156	5.08	4.783	0.0
	Max	112.0	6.277	5.986	104.338	30.897

## Appendix C: Priors for Bayesian hierarchical generalized linear-mixed models

**Table 18:** Overview of model priors

Log-linear regression model	Logistic regression model
$\beta _0\sim \mathcal {N}(6, 1.5)$	$\beta _0\sim \mathcal {N}(0, 4)$
$\beta _1\dots \beta _4 \sim \mathcal {N}(0,1)$	$\beta _1\dots \beta _4 \sim \mathcal {N}(0,1)$
$$\sigma \sim \mathcal {N}(0, 1)$$	

## Footnotes

1 https://artificialintelligenceact.eu/

2 Typically, the vocabulary is composed of a set of sub-word tokens which allow for greater flexibility in representing rare or unseen words (Kudo & Richardson, 2018; Sennrich et al., 2016). For simplicity, in this section, we refer to these sub-word tokens as words.

3 Following pre-training and supervised instruction-tuning, models can be further adapted with additional post-training strategies to “align” the model’s predictions with human preferences (Ouyang et al., 2022; Rafailov et al., 2024; Ethayarajh et al., 2024; Gheshlaghi Azar et al., 2024; Xu et al., 2024) and to incentivize complex reasoning processes (DeepSeek-AI et al., 2025).

4 When $l = 1$, beam search is equivalent to greedy search.

5 Note that $k = 1$ is equivalent to greedy search while $k = \left| V\right|$ is equivalent to ancestral sampling.

6 https://www.microsoft.com/en-us/research/blog/phi-2-the-surprising-power-of-small-language-models/

7 https://huggingface.co/mistralai/Mistral-7B-Instruct-v0.1

8 https://huggingface.co/WizardLM/WizardLM-13B-V1.2

9 At the time of model prompting, beam search was not implemented for Phi-2.

10 Number of tokens refers to sub-word tokens, not words.

11 Within the eye movements in reading research community, many research questions are formulated on the word level, which is why we define word-based areas of interest and map fixations as well as model internals (attention scores, transition scores, hidden states) to the word level. However, some research questions require analyses on the character level, which is why we also provide the files designating the character-level areas of interest for full flexibility.

12 https://pypi.org/project/wordfreq/

13 https://github.com/LuminosoInsight/exquisite-corpus

14 https://pypi.org/project/py-readability-metrics/

15 https://spacy.io/

16 https://www.sr-research.com/eyelink-portable-duo/

17 Note that we enumerate the words across all stimulus texts such that each word of each stimulus text has its own index.

18 https://github.com/DiLi-Lab/EMTeC/

19 https://osf.io/ajqze/

20 The transition scores amount to around 32 GB; the hidden states to 114 GB; and the attention scores to 193 GB.

21 https://dataverse.harvard.edu/dataset.xhtml?persistentId=doi:10.7910/DVN/GCU0W8

22 Eye-tracking corpora with naturalistic stimuli can be leveraged for researching a variety of psycholinguistic phenomena; they allow for evaluating theories without first explicitly designing an experiment.

## Declarations

### Code availability

The code to reproduce the stimulus generation, data pre-processing, and analyses is available via the GitHub repository https://github.com/DiLi-Lab/EMTeC/.

### Open Practices Statement

Materials and code are available in a GitHub repository at https://github.com/DiLi-Lab/EMTeC/. The eye-tracking data files are stored in an OSF repository at https://osf.io/ajqze/, but can be downloaded via the GitHub repository. The tensors are stored in a Harvard Dataverse at https://dataverse.harvard.edu/dataset.xhtml?persistentId=doi:10.7910/DVN/GCU0W8 and can also be downloaded via the GitHub repository. None of the reported studies were preregistered.
